# Discovery of Exosomes From Tick Saliva and Salivary Glands Reveals Therapeutic Roles for CXCL12 and IL-8 in Wound Healing at the Tick–Human Skin Interface

**DOI:** 10.3389/fcell.2020.00554

**Published:** 2020-07-16

**Authors:** Wenshuo Zhou, Faizan Tahir, Joseph Che-Yen Wang, Michael Woodson, Michael B. Sherman, Shahid Karim, Girish Neelakanta, Hameeda Sultana

**Affiliations:** ^1^Department of Biological Sciences, Old Dominion University, Norfolk, VA, United States; ^2^Center for Molecular and Cellular Biosciences, School of Biological, Environmental, and Earth Sciences, The University of Southern Mississippi, Hattiesburg, MS, United States; ^3^Department of Molecular and Cellular Biochemistry, Indiana University, Bloomington, IN, United States; ^4^Electron Microscopy Center, Indiana University, Bloomington, IN, United States; ^5^Department of Biochemistry and Molecular Biology, University of Texas Medical Branch at Galveston, Galveston, TX, United States; ^6^Sealy Center for Structural Biology and Molecular Biophysics, University of Texas Medical Branch at Galveston, Galveston, TX, United States; ^7^Center for Molecular Medicine, Old Dominion University, Norfolk, VA, United States; ^8^Division of Infectious Diseases and International Health, Department of Medicine, University of Virginia School of Medicine, Charlottesville, VA, United States

**Keywords:** exosomes, ticks, saliva, salivary glands, interleukin-8 (IL-8), C-X-C motif chemokine ligand 12 (CXCL12), skin barrier, wound healing

## Abstract

Ticks secrete various anti-coagulatory, anti-vasoconstrictory, anti-inflammatory, and anti-platelet aggregation factors in their saliva at the bite site during feeding to evade host immunological surveillance and responses. For the first time, we report successful isolation of exosomes (small membrane-bound extracellular signaling vesicles) from saliva and salivary glands of partially fed or unfed ixodid ticks. Our data showed a novel role of these *in vivo* exosomes in the inhibition of wound healing *via* downregulation of C-X-C motif chemokine ligand 12 (CXCL12) and upregulation of interleukin-8 (IL-8). Cryo-electron microscopy (cryo-EM) analysis revealed that tick saliva and salivary glands are composed of heterogeneous populations of *in vivo* exosomes with sizes ranging from 30 to 200 nm. Enriched amounts of tick CD63 ortholog protein and heat shock protein 70 (HSP70) were evident in these exosomes. Treatment of human skin keratinocytes (HaCaT cells) with exosomes derived from tick saliva/salivary glands or ISE6 cells dramatically delayed cell migration, wound healing, and repair process. Wound healing is a highly dynamic process with several individualized processes including secretion of cytokines. Cytokine array profiling followed by immunoblotting and quantitative-PCR analysis revealed that HaCaT cells treated with exosomes derived from tick saliva/salivary glands or ISE6 cells showed enhanced IL-8 levels and reduced CXCL12 loads. Inhibition of IL-8 or CXCL12 further delayed exosome-mediated cell migration, wound healing, and repair process, suggesting a skin barrier protection role for these chemokines at the tick bite site. In contrast, exogenous treatment of CXCL12 protein completely restored this delay and enhanced the repair process. Taken together, our study provides novel insights on how tick salivary exosomes secreted in saliva can delay wound healing at the bite site to facilitate successful blood feeding.

## Introduction

Ticks and tick-borne diseases are of major concern due to medical and veterinary health importance (Nuttall et al., 2000; Neelakanta and Sultana, 2015; de la Fuente, 2018). Ticks not only cause damage/inflammation to human or animal skin but also facilitate transmission of deadly pathogens to the host (Wikel and Whelen, 1986; Wikel, 1996a, 2018; Nuttall et al., 2000; Nuttall and Labuda, 2003, 2004; Bernard et al., 2015). In these triad interactions that involve pathogen, arthropod, and vertebrate/human host, ticks serve as medically important vectors for pathogen transmission to the host (Wikel and Whelen, 1986; Labuda et al., 1996; Nuttall et al., 2000; Nuttall and Labuda, 2004; Neelakanta et al., 2010; Sultana et al., 2010; Leitner et al., 2011; Wikel, 2018). Several studies have shown that during blood feeding, ticks transmit a variety of pathogens that mainly includes bacteria from rickettsial family and Lyme disease agent and viruses such as tick-borne encephalitis virus (TBEV) that belongs to Flaviviridae family (Wikel and Whelen, 1986; Labuda et al., 1996; Nuttall et al., 2000; Nuttall and Labuda, 2004; Neelakanta et al., 2010; Sultana et al., 2010; Leitner et al., 2011; Wikel, 2018; Zhou et al., 2018). We recently reported for the first time that tick-borne Langat virus (LGTV), a model pathogen closely related to TBEV, is transmitted *via* infectious tick exosomes containing viral RNA and proteins (Zhou et al., 2018). Our study also showed that LGTV profusely used arthropod exosomes that were capable of transmission of infectious viral RNA and proteins to the HaCaT cells and human blood endothelial cells (Zhou et al., 2018). Keratinocytes are the initial and highly critical defense barrier lining of the human skin that comes in direct contact during the bites from infected ticks (Hanel et al., 2013; Kim et al., 2014; Zhou et al., 2018). Keratinocytes react by producing the pro-inflammatory chemokines like interleukin-8 (IL-8) to recruit leukocytes such as neutrophils at the site of damage caused by pathogenic viruses, bacteria, fungi, parasites, UV radiation, heat, or water loss (Kamata et al., 2011; Jiang et al., 2012; Hanel et al., 2013; Kim et al., 2014; Scholl et al., 2016; Zhou et al., 2018). Other than keratinocytes, skin harbors several residential immune cells that check and limit infections by secreting inflammatory cytokines, type I interferons (IFNs), critical chemokines, and antimicrobial peptides or by directly killing pathogens with alternative mechanisms such as synthesis of nitric oxide or reactive oxygen species (ROS) (Labuda et al., 1996; Labuda and Randolph, 1999; Nuttall and Labuda, 2003; Hanel et al., 2013; Bernard et al., 2014, 2015; Scholl et al., 2016; Glatz et al., 2017; Han et al., 2017; Zhou et al., 2018; Nuttall, 2019). Studies addressing transcriptional regulation, immunohistochemistry, and immune cells targeting the tick–virus–host interface during early stages of infections have highlighted participation of inflammatory cytokines and chemokines along with infiltration of mononuclear cells and neutrophils (Labuda et al., 1996; Labuda and Randolph, 1999; Hanel et al., 2013; Bernard et al., 2014, 2015; Hermance and Thangamani, 2015; Zhou et al., 2018). Although skin keratinocytes constitute an efficient physical and immune barrier at the vector and pathogen interface, ticks have evolved appealing mechanisms to thwart and evade the innate and adaptive immune responses at this region (Wikel, 1996a,b, 2018; Bowman et al., 1997; Nuttall et al., 2000; Nuttall and Labuda, 2003, 2004; Brossard and Wikel, 2004; Leitner et al., 2011; Hanel et al., 2013; Bernard et al., 2014, 2015; Kotal et al., 2015; Nuttall, 2019). In order to allow the firm attachment, acquire a continuous blood meal, successfully complete feeding and repletion, ticks do secrete a combination of pharmacologically bioactive salivary factors into this feeding site (Wikel and Whelen, 1986; Labuda et al., 1996; Wikel, 1996a; Nuttall and Labuda, 2003; Lewis et al., 2015; Scholl et al., 2016; Vora et al., 2017; Bakshi et al., 2018). Some of these tick salivary factors include the inhibitors of the itch and/or pain or in combination of both, anticoagulants, immunomodulators, vasodilators (that dilate blood vessels), and antiplatelet molecules (that can avoid platelet-mediated killing mechanisms) (Wikel and Whelen, 1986; Labuda et al., 1996; Wikel, 1996a; Bowman et al., 1997; Nuttall and Labuda, 2003, 2004; Bernard et al., 2015; Kotal et al., 2015; Lewis et al., 2015; Scholl et al., 2016; Glatz et al., 2017; Vora et al., 2017; Bakshi et al., 2018; Nuttall, 2019).

Numerous studies have shown that several tick salivary factors play differential roles to facilitate evasion of host immune responses (Bowman et al., 1997; Nuttall and Labuda, 2004; Bernard et al., 2015; Kotal et al., 2015; Glatz et al., 2017; Nuttall, 2019). We have shown that tick salivary factors elicit differential levels of fibrinogenolysis upon blood feeding on immunocompetent or immunodeficient mice (Vora et al., 2017). A differential level of D-dimer, a fibrin degradation product, was noted in ticks fed on different immune background animals. This work defined a novel role for tick heat shock protein 70 (HSP70)-like molecule(s) in contributing to these differential levels of fibrinogenolytic activity (Vora et al., 2017). Upon feeding on immunodeficient animals, one of the transcripts of HSP70-like protein was reduced, suggesting a requirement for this factor in tick saliva (Vora et al., 2017). Independent work from our laboratory also showed the presence of HSP70 as an enriched protein in ISE6 tick cell-derived exosomes (Zhou et al., 2018). The presence of HSP70 in tick exosomes suggested that salivary factors are perhaps secreted *via* exosomes. We now provide first evidence for the successful isolation of *in vivo* exosomes from Ixodidae tick saliva and salivary glands and show that these exosomes inhibit tissue healing *via* host molecules like IL-8 and C-X-C motif chemokine ligand 12 (CXCL12). Furthermore, cytokine array profiling of human keratinocytes incubated with tick saliva/salivary glands or ISE6 cell-derived exosomes revealed a skin barrier protection role for secreted IL-8 and CXCL12. The present study strongly suggests that tick exosomes are perhaps the carriers of bioactive salivary factors that facilitate blood feeding and pathogen transmission.

## Materials and Methods

### Ticks and Other Animals

The Gulf Coast tick, *Amblyomma maculatum* (*Am*) was maintained at the University of Southern Mississippi according to established methods (Patrick and Hair, 1975). Unfed *Am* adult female ticks were purchased from Oklahoma State University’s tick-rearing facility (Stillwater, OK, United States). Female *Am* were kept at room temperature with approximately 90% relative humidity under a photoperiod of 14 h of light and 10 h of darkness before infestation on sheep. Female *Am* were blood-fed on sheep and removed at intervals between 1 and 11 days, depending upon the experimental protocol. Adult unfed female-blacklegged ticks, *Ixodes scapularis* (*Is*), were obtained from BEI Resources [American Type Culture Collection (ATCC)] and were maintained at our laboratory in a similar way as described above. The unfed female *Is* were used as an independent group and not to compare with the partially fed group of female *Am*.

### Collection of Tick Saliva, Salivary Glands, and Tick Cell Culture Supernatants

Saliva and salivary glands were collected from female *Am* as described (Karim et al., 2002, 2011). Briefly, partially fed female *Am* were stimulated to salivate into capillary tubes by induction method and with 1% pilocarpine buffered in 1 × phosphate buffered saline (PBS) (Ribeiro et al., 2004; Crispell et al., 2019). Freshly collected saliva pooled from 100–200 *Am* female ticks was immediately stored at −80°C or until subsequent exosome purifications were performed. Partially fed female *Am* were processed for isolation of salivary gland tissues (pooled from 80–100 separate batch of *Am*) that were dissected within 2 h of removal of these ticks from sheep (Karim et al., 2002, 2011). *Am* salivary glands were washed in M-199 buffer and stored in 0.15 M Tris-HCl, pH 8.0, containing 0.3 M NaCl, 10% glycerol, and 1% protease inhibitor cocktail (Amresco, Solon, OH, United States) and at −80°C. In addition, salivary glands were also dissected from unfed female *Is* (pooled from 80–100 ticks) and collected in 1 × PBS containing protease or phosphatase cocktail and stored immediately at −80°C. These two groups of salivary glands from *Am* or *Is* are not for comparison but were used as independent samples to show the presence of exosomes and the detection of exosomal markers. As we have successfully isolated exosomes from *I. scapularis* ISE6 tick cell line in our previous study, we used these cells as a positive control for isolation of purified exosomes (14). ISE6 cells were grown in L15300 media as per the culture methods (Zhou et al., 2018). Tick cell (2 × 10^6^) culture supernatants were collected and processed for isolation of exosomes as described in our previous study (Zhou et al., 2018). The media used for tick cells are free of endotoxins, and we do not expect any endotoxins in our exosome preparations.

### Exosome Isolation From Tick Saliva, Salivary Glands, and Concentrated ISE6 Cell Culture Supernatants

OptiPrep density gradient exosome (DG-Exo) isolation or differential ultracentrifugation methods were used for isolation and purification of exosomes (Thery et al., 2006; Zhou et al., 2018). Details for these procedures are schematically shown in our previous studies (Vora et al., 2018; Zhou et al., 2018). To isolate exosomes, we used concentrated pooled saliva from several partially fed female *Am* ticks. To minimize the cell damage, *Am* or *Is* salivary glands were gently processed for exosome isolation. We successfully isolated *in vivo* exosomes as six different fractions using DG-Exo-isolation method (Vella et al., 2017) for soft tissues such as brain. We included C57BL/6 mouse brain slices (20 mg) as internal control along with tick salivary glands (80–100 pairs, weight ∼20 mg) to isolate six different exosome fractions. Frozen brain tissues were sliced lengthwise with razor blade to generate small sections that were kept frozen. Tissues (salivary glands and brain slices) were weighed (to similar weight) while partially frozen and transferred to a tube containing 75 U/ml of collagenase type 3 in Hibernate-E medium (at a ratio of 800 μl per 100 mg of tissue). Tissues were incubated for a total of 20 min in a shaking water bath at 37°C and were gently mixed by inversion after 5 min, then returned to the incubation, and pipetted for several times after 5–10 min and again kept for the remaining time. After the incubations, tissues were immediately kept on ice, with the addition of protease and phosphatase inhibitors cocktail. To separate clear supernatants, disassociated tissue was spun at 300 *g* for 5 min followed by transfer and centrifugation of the supernatant at 2,000 *g* for 10 min, and again 10,000 *g* for 30 min (all spins were done at 4°C). Tick cell culture supernatants as positive control (∼20 ml) were collected and centrifuged at 4°C (480 *g* for 10 min followed by 2,000 *g* for 10 min to remove cell debris and dead cells). Cell culture supernatants were then concentrated to volume of 2–2.5 ml using the Corning Spin-X UF concentrators or centrifugal filter device with a 5 k nominal molecular weight limit (NMWL). The concentrated tick culture supernatants were processed for OptiPrep^TM^ (DG-Exo) isolation as described (Zhou et al., 2018). Briefly, discontinuous gradients of 40% (w/v), 20% (w/v), 10% (w/v), and 5% (w/v) solutions of iodixanol were prepared from the stock solution of OptiPrep^TM^ 60% (w/v) of aqueous iodixanol (Axis-Shield PoC, Norway) with 0.25 M Sucrose/10 mM Tris, pH 7.5. Concentrated saliva, salivary gland suspensions, or tick cell culture supernatants were overlaid onto the top of the gradient and centrifuged at 100,000 *g* for 18 h at 4°C. Six individual fractions of ∼3 ml were collected (from top to bottom, as fractions 1–6, and with increasing density) and diluted with 5 ml of sterile PBS. Fractions were centrifuged again at 100,000 *g* for 3 h at 4°C and followed by one more wash with 5 ml of PBS and resuspended in 80–200 μl of PBS. Purified DG-Exo preparations were stored at −80°C and used later for further analysis. DG-Exo-isolation was used for generation of six fractions of exosomes that were used in immunoblotting analysis. In addition, exosomes from saliva and tick cell culture supernatants were isolated by differential ultracentrifugation method as described in our studies (Vora et al., 2018; Zhou et al., 2018, 2019). To isolate exosomes without a mixture of bovine exosomes, we plated ISE6 cells (1 × 10^6^ cells, as six replicates) in exosome-free fetal bovine serum [Exo-free FBS obtained from Systems Biosciences Inc. (SBI)]. To perform sonication, exosomes were sonicated on ice five times with each time period of 20 s and with a 20-s cooling break in between (20-s bursts with 20-s resting periods). After sonication of exosomes, they were freeze and thawed at −80°C three times. Sonicated or not sonicated groups of exosomes were run on native polyacrylamide gel electrophoresis (PAGE) (Zhou et al., 2018) to confirm the lysis of sonicated/treated exosomes. Furthermore, sonicated or not sonicated groups of exosomes were treated with 5 mg/ml of RNaseA/DNaseI mixture for 15 min at 37°C, followed by RNA extractions and quantitative real-time PCR (QRT-PCR) analysis to reveal the cytokine loads. For the measurement of exosome numbers, we used the microfluidic resistive pulse sensing (MRPS) analysis. MRPS was performed with nCS1 Spectradyne nanoparticle analyzer (Spectradyne LLC, United States) and by using TS-300 filter that measures particles from 50 to 300 nm. Exosome measurements were performed as three independent replicates.

### Cryo-Electron Microscopy

Cryo-electron microscopy (cryo-EM) was performed at two independent institutional facilities [University of Texas Medical Branch (UTMB) and Indiana University (IU)] on *Am* saliva or salivary gland-derived exosomes. At UTMB facility, exosomes were vitrified on carbon holey film grids (Sherman et al., 2006, 2009) (R2 × 2 Quantifoil; Micro Tools GmbH, Jena, Germany; or C-flat^TM^, Protochips, Raleigh, NC, United States). Briefly, purified concentrated suspensions of exosomes in PBS were applied to the holey films in a volume of ca. 3 μl, blotted with filter paper, and plunged into liquid ethane cooled in a liquid nitrogen bath. We used computerized Vitrobot plunger (FEI, Hillsboro, OR, United States) for freezing. Frozen grids were stored under liquid nitrogen and transferred to a cryo-specimen holder (70 deg. 626, Gatan, Inc., Pleasanton, CA, United States or 2550 cryo-tomography holder, E.A. Fischione Instruments, Inc., Export, PA, United States) under liquid nitrogen before loading into a JEOL 2200FS or a JEOL 2100 electron microscope (JEOL Ltd., 3-1-2 Musashino, Akishima, Tokyo 196-8558, Japan). JEOL 2200FS was equipped with in-column energy filter (omega type) and a field emission gun (FEG); JEOL 2100 had a LaB_6_ filament, both were operated at 200 keV. Grids were maintained at near-liquid nitrogen temperature (−172 to −180°C) during imaging. Preliminary screening and imaging of exosomes were done using a 4k × 4k Gatan US4000 CCD camera (Gatan, Inc., Pleasanton, CA, United States), and final imaging was done at indicated 40,000 × magnification with a 5k × 4k Direct Electron Detector camera (DE-20, Direct Electron, Inc., San Diego, CA, United States) using a low-dose imaging procedure. An in-column omega electron energy filter was used during imaging with a zero-loss electron energy peak selected with a 20-eV slit. Images were acquired with a ca. 20 electrons/Å^2^ dose; the pixel size corresponded to 1.5 Å on the specimen scale. We used a 2.0–2.3-μm defocus range for imaging. For cryo-EM analysis at IU facility, 4 μl of sample solution was applied on a glow-discharged holey carbon film coated copper grid with an additional layer of ultrathin carbon film (Quantifoil). The grid was quickly frozen using FEI Vitrobot^TM^ Mark III with 2.5-s blotting time, at 8°C, and under 100% humidity. Frozen-hydrated specimen was transferred using Gatan 626 cryo-holder and imaged by JEM-1400plus. Images were screened and taken under low-dose conditions on a Gatan OneView camera. Overall, exosome images were acquired from two independent batches of exosomes (from *Am* saliva and salivary glands) at each institution. For cryo-EM imaging, we performed two independent experiments at each facility.

### Immunoblotting Analysis and ELISA Assay

For immunoblotting assays, exosomal fractions (1–6) were prepared by DG-Exo-isolation method and resuspended in freshly prepared cold 1 × PBS and processed for detection of exosomal-enriched molecules. Exosomal proteins isolated from brain tissue, tick saliva, or salivary glands were measured by bicinchoninic acid (BCA) method and at 562 nm. Equal volumes (20 μl) of each exosomal fraction (1–6) were loaded onto 12% sodium dodecyl sulfate (SDS)-PAGE gels, followed by immunoblotting and labeling with specific exosomal markers such as HSP70 (rat monoclonal; Cell Signaling Technologies, Inc.) or CD63 (mouse monoclonal; Santa Cruz Biotechnologies, Inc.) antibodies. Both HSP70 and CD63 antibodies were used at 1:500 dilutions. BCA assay was performed on exosomal protein amounts from *Am* saliva or salivary glands, *Is* salivary glands, or ISE6 cell culture supernatants, or mice brain tissue using Pierce kit and by following manufacturer’s instructions. The BCA assay was performed in triplicate. For multiple human cytokine array Panel A (Proteome Profiler Array Kit, Catalog Number ARY005 purchased from R&D Systems, Biotechne) that identifies the cytokines and chemokines that are differentially regulated upon secretion, blots were incubated with cell culture supernatants (0.5 ml) from HaCaT cells treated (for 24 h) with tick exosomes derived from *Am* saliva or salivary glands, *Is* salivary glands, or ISE6 cell culture supernatants. Briefly, expression levels of 36 human cytokines/chemokines secreted protein levels were analyzed and were processed as per the instructions from the manufacturer, and the assay details are provided on the company’s manual. The human cytokine proteome profiler array consists of four independent nitrocellulose membranes spotted with carefully selected capture antibodies for 36 different cytokines/chemokines, including three pairs of positive spots and one pair of negative spot.

To validate the cytokine array, we performed immunoblotting analysis and loaded 20 μl of concentrated culture supernatants (concentrated using the Corning Spin-X UF concentrators or centrifugal filter device with a 5 k NMWL) collected from HaCaT cell treated (24 h) with respective groups of tick exosomes on to 12% SDS-PAGE gels, followed by probing with IL-8 (Santa Cruz Biotechnologies, Inc.) or CXCL12 (Cell Signaling Technologies, Inc.) mouse monoclonal antibodies, respectively. For immunoblotting analysis to detect the presence of CD63 in *Am* salivary gland whole lysates or ISE6 whole cell lysates (20 μg) or ISE6 cell-exosomal lysates (10 μg), samples were loaded on to 12% SDS-PAGE gel. Respective secondary antibodies (Santa Cruz Biotechnologies, Inc.) with horseradish peroxidase (HRP) conjugates (dilutions of 1:5,000) were used for each primary antibody. Total protein profiles (images obtained from stain-free gels) served as loading controls. Antibody binding was detected with WesternBright ECL kit (Advansta, BioExpress). Membranes were exposed for a number of seconds depending on the intensity of the signals or until the desired signal is reached. Blots were imaged using Chemidoc MP imaging system and processed using ImageLab software from the manufacturer (Bio-Rad). All immunoblotting analysis was performed in duplicates. For ELISA, we coated Nunc grade plates as six replicates with 200 μl (each well) of cell culture supernatants obtained from HaCaT cells treated (24 h) with tick exosomes derived from *Am* saliva or salivary glands or ISE6 cells overnight. Samples were blocked with 3% bovine serum albumin (BSA) for 2 h at 4°C, followed by IL-8 primary antibody (1:100 dilution) incubation for 1 h at 4°C, and HRP-conjugated secondary antibody (1:2,500 dilution) incubation for 1 h at room temperature (RT). SureBlue TMB Microwell Peroxidase Substrate and Stop solution (from KPL) were used according to manufacturer’s instructions. Optical density was measured at an absorbance of 450 nm using a Multimode infinite M200 Pro Microplate reader (Tecan). ELISA was performed in six replicates for each group.

### Wound Healing Assays and Phase Contrast Microscopy

HaCaT cells were grown in complete Dulbecco’s modified Eagle’s medium (DMEM) containing 5% heat-inactivated FBS (Invitrogen, GIBCO; Thermo Scientific) and maintained as per the ATCC Company guidelines. Briefly, 1–1.5 × 10^6^ HaCaT cells were plated in six-well plates with regular media, and after overnight attachment, culture medium was removed, and fresh medium with 10% FBS was added to these cells. Before exosome treatments, images of HaCaT cell monolayers were taken as before scratch (also referred as before scratch). Wounds/scratches were generated in the middle of the HaCaT cell monolayers and by using sterile 1-ml blue pipette tips (nearly 0.5–0.75 mm). Monolayers of HaCaT cells were then treated for 24 h (at 37°C) with purified exosomes from *Am* saliva/salivary glands or *Is* salivary gland or ISE6 cell-derived exosomes. Cells were monitored, and several phase contrast images were collected (using EVOS FL system, Thermo Scientific, at 10 × magnification, and with a scale bar of 400 μm) at different time points (of 0, 4, 8, 16, and 24 h) after scratches from each group. HaCaT monolayers without exosome treatments but with scratches were considered as untreated (UT) controls in each assay. Images of HaCaT cell monolayers obtained immediately after treatment (for 24 h) with exosomes from different groups are considered as 0 h. To determine the role of tick exosomes on the cell migration and wound repair process, we continuously collected images at the indicated time points of 4, 8, 16, and 24 h post wound generation. Representative images are shown in each panel. For antibody or protein treatments, HaCaT cells were plated (as above) overnight, changed to fresh DMEM medium with 10% FBS and treated for 12 h with either IL-8 or CXCL12 or both antibodies or IgG isotype control antibody (2 μg per well/per group, for each antibody; IL-8 or CXCL12 or IgG isotype) or treated for 4 h with purified CXCL12-glutathione *S*-transferase (GST)-tagged protein or GST protein alone (2 μg per well/per group, for each protein; CXCL12 or GST) at 37°C, followed by scratches to generate wounds and treatments with tick exosomes of the respective groups. Scratched monolayers were treated (24 h) with exosomes, and images were obtained at the indicated time points as described above. IL-8 (B-2) antibody (Catalog number: sc-8427) and CXCL12/stromal cell-derived factor 1α (SDF-1α) (F-4) antibody (Catalog number: sc-518066) were obtained from Santa Cruz Biotechnology, Inc. The relevant isotype control IgG antibody (Catalog number: ab37355) was purchased from Abcam. We used CXCL12 or SDF-1α (hBA-68) (Catalog number: sc-4654) purified protein produced from *Escherichia coli* bacterial lysates (> 98%) and supplied as 35-kDa biologically active, GST-tagged fusion protein corresponding to 68 amino acids of the SDF-1α of human origin (Santa Cruz Biotechnology, Inc.). CXCL12 purified protein used in this study is free of endotoxin and is obtained from a commercial vendor. GST purified protein was produced in our laboratory from *E. coli.* For siRNA studies, monolayers of HaCaT cells were transfected with ∼1 μg of IL-8 siRNA for 24 h (and as per the manufacturer’s instructions; Santa Cruz Biotechnologies, Inc.), followed by wound healing assays (for another 24 h) as described above. Scrambled siRNA serves as a control to monitor transfection efficiency. UT internal controls were considered for wound healing assays. All assays were performed in duplicate for each group of tick exosomes, and data from one independent experiment is shown. Quantitative estimation as percentages of remaining wound sizes against different time points after scratching was calculated using ImageJ (NIH generated imaging software) and by measurement of wound closures in each group and in each independent experiment in comparison to their respective UT controls. Wounds at 0 h were considered as 100% for all groups. Bar graphs were generated using GraphPad Prism 6 software.

### RNA Extraction, cDNA Synthesis, PCR and QRT-PCR Analysis

Briefly, HaCaT cells were seeded (5 × 10^5^) overnight in 5% FBS containing DMEM medium. Next day, cells were changed to fresh DMEM medium containing 10% FBS. HaCaT cell monolayers were treated with tick exosomes from respective groups for 24 or 72 h posttreatment. Freshly prepared exosomes (by DG-Exo-isolation method) were collected in PBS, pooled, and stored frozen at −80°C for treatment of HaCaT cells. Total RNA from HaCaT cells was extracted (at either 24 or 72 h) using Aurum Total RNA Mini kit (Bio-Rad) and following manufacturer’s instructions. During RNA extractions, DNaseI treatment was performed as on column digestion and as per the recommendations from the manufacturer. Using Bio-Rad iScript cDNA synthesis kit, 1 μg RNA was converted to cDNA and used as template for the amplification and determination of cytokine expression by QRT-PCR. Arrays of cytokines were analyzed at both tested time points of 24 and 72 h posttreatment. Independent UT control groups were maintained for each cytokine group analyzed in this study. Human *beta-actin* amplicons were quantified with published primers (Sultana et al., 2010, 2012) and used for normalization. QRT-PCR was performed using iQ-SYBR Green Supermix (Bio-Rad, United States). Standard curves were prepared using 10-fold serial dilutions starting from standard 1 to 6 of known quantities of *actin* or different cytokine gene fragments. UT samples served as internal controls. For amplification of CD63-like molecules, we used female *Is* or ISE6 cells extracted RNA that is converted to cDNA as template in QRT-PCR analysis. Following are the primer sequences used for CD63-like molecules amplifications: for ISCW001785 (forward primer 5′ GCATCCTCCACATCTTCAACTTCA 3′ and reverse primer 5′ CAGGAGCAGGAGGAAGGTGT 3′), for ISCW010731 (forward primer 5′ GCTCTGGGAAGAGTGTGCGT 3′ and reverse primer 5′ CCTCCTGGCTTTTGAGTCCTCT 3′), and for ISCW014150 (forward primer 5′ GGCAGGCATTTTGGGATT CA 3′ and reverse primer 5′ GCAGCACGAGAAAGGGACA 3′). The primers for cytokines and chemokines were used from published studies (Strausberg et al., 2002; Cho et al., 2007; Miyamoto et al., 2007; Schutyser et al., 2007; Kang et al., 2009; Sultana et al., 2009; Xie et al., 2009; Kawane et al., 2010; Carneiro et al., 2011; Bernard et al., 2016; Roy et al., 2017) and shown in Supplementary Table S3. For IL-8 siRNA analysis, after acquiring the images for wound healing assay, HaCaT cells were processed for RNA extractions followed by PCR amplification of IL-8 and actin. We loaded PCR samples on 1.2% DNA agarose gel for detecting the IL-8 and actin loads. For all RNA extractions, we have considered six replicates and performed the QRT-PCR in duplicate for each replicate.

### Statistics

Statistical differences observed in data sets were analyzed using GraphPad Prism6 software and Microsoft Excel. The non-paired, two-tail Student *t*-test was performed (for data to compare two means) for the entire analysis. Error bars represent mean (+SD) values, and *P*-values of <0.05 were considered significant in all analyses. Statistical test and *P*-values are indicated for significance. We have also performed the two-way ANOVA without replicates analysis to measure the significant differences in wound healing percentages and treatments.

### Ethics Statement

All animal experiments were conducted in strict accordance with the recommendations in the Guide for the Care and Use of Laboratory Animals of the National Institutes of Health (NIH). Protocol for performing the blood feeding of *Am* ticks on sheep was approved by the Institutional Animal Care and Use Committee (IACUC) at the University of Southern Mississippi (protocol # 15101501). All efforts were made to minimize animal sufferings. For both ticks and human cell line experiments, we used the institutionally approved protocol #15-014.

## Results

### Cryo-Electron Microscopy Analysis Revealed a Novel Discovery of Exosomes in Tick Saliva and Salivary Glands

**FIGURE 1:**
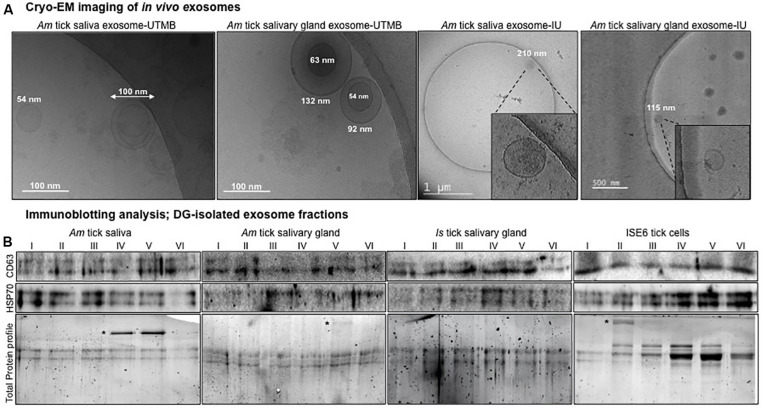
Isolation of *in vivo* exosomes derived from tick saliva and salivary glands and detection of exosomal markers. **(A)** Cryo-electron microscopy (Cryo-EM) images collected from two independent institutions [University of Texas Medical Branch (UTMB) and Indian University (IU)] showing exosomes isolated from *Am* saliva or salivary glands. Scale bar indicates 100 nm, or 1 μm, or 500 nm, respectively. For better estimation, scales have been provided close to individual exosomes to show the heterogeneous population of exosomes. For images from IU, insets show details of exosome structures from the field. **(B)** Immunoblotting analysis showing detection of exosomal-enriched markers CD63 and HSP70 in six different exosome fractions derived from *Am* saliva or salivary glands, *Is* salivary glands or ISE6 cells. CD63 and heat shock protein 70 (HSP70) levels indicate the presence and enrichment of tick exosomal markers in each fraction. *Am* represents *Amblyomma maculatum*, *Is* indicates *Ixodes scapularis*, and ISE6 denotes *Is-*derived cell line. Total protein profile images of stain-free gels shown for each group serve as a loading control. Asterisk indicates additional exosomal-enriched proteins detected in the gel images.

In this study, we have presented a novel discovery showing the presence of *in vivo* exosomes/extracellular vesicles (EVs) in tick saliva and salivary gland tissues. Our cryo-EM analysis showed the presence of purified tick saliva or salivary gland-derived *in vivo* exosomes with the size ranges of 30–200 nm in diameter (Figure 1A) that are similar to exosomes isolated from ISE6 cells or other mammalian cells (Fevrier and Raposo, 2004; Ludwig and Giebel, 2012; Regev-Rudzki et al., 2013; Zhou et al., 2018, 2019). Exosomes isolated from tick saliva or salivary glands showed a heterogeneous population of vesicles with variable sizes (Figure 1A). We observed no differences in exosomes imaged from either saliva or salivary glands and neither from two independent institutional research groups (Figure 1A). Additional cryo-EM representative images are shown from both independent groups for further confirmation that *in vivo* tick exosomes are intact vesicles with membrane-bound lipid bilayers (Supplementary Figure S1A). Furthermore, measurement of exosome numbers by MRPS analysis revealed that *Am* saliva-derived exosome sizes (measured as particle diameter against concentration of particles) were variable from 50 to 110 nm (Supplementary Figure S1B). Particle diameter vs. transit time (time taken to move particles in the thin capillaries of Spectradyne device) was also calculated by excluding events that serve as background noise and/or false positives (Supplementary Figure S1B). Only included events represent particles considered for this analysis (Supplementary Figure S1B). Fewer particles were counted due to less number/concentration (±2.18E + 08, 1.17E + 08/ml) of exosomes in *Am* saliva samples that were diluted with PBS/Tween 20 (PBST) in a ratio of 1:1 (Supplementary Figure S1B). In contrast to saliva-derived exosomes, we counted (±3.99E08, 3.55E + 08/ml) higher number of *Is* salivary gland-derived exosomes (also diluted with PBST in a 1:1 ratio) that were variable between 50 and 180 nm in sizes (Supplementary Figure S1C). Similar to data shown for *Am* saliva-derived exosomes, we considered only the included events for particle counts in salivary gland-derived exosomes (Supplementary Figure S1C).

### Detection of Enriched Exosomal Markers in Tick Saliva and Salivary Gland-Derived Exosomes Confirmed the Presence of Extracellular Vesicles in These *in vivo* Samples

Next, we collected exosomes as six different fractions from saliva or salivary glands of partially fed *Am* or salivary glands from unfed *Is* female ticks or ISE6 cells or soft brain tissue as internal control (Vella et al., 2017). CD63 (a specific exosomal marker in mammalian cells) was highly enriched in exosomal fractions isolated (resuspended in 20 μl of PBS) from brain tissue samples (Supplementary Figure S2A). The protein profile image serves as a loading control (Supplementary Figure S2A). Exosomal protein measurement by BCA method showed a linear graph and the standards *R*^2^ value to be close to 1 (Supplementary Figure S2B). The *R*^2^ value generated from BSA standards is shown for estimation. Exosomal protein amounts were determined to be 0.39 μg/μl (∼8 μg per 20 μl) for each tick group (Supplementary Figure S2C). Immunoblotting analysis with 20 μl of each tick exosomal fraction (collected by DG-Exo isolation) revealed the presence of HSP70 (70 kDa) from both *Am* saliva and salivary glands (Figure 1B). In addition, we found abundant amounts of HSP70 in *Is* salivary gland-derived exosomal fractions (Figure 1B). As expected, HSP70 was also detected in ISE6 cell-derived exosomal fractions (Figure 1B). We found enhanced levels of HSP70 in ISE6 cell-derived exosomes when compared to saliva or salivary gland-derived exosomes, suggesting that higher exosomal numbers are derived from a large volume of tick cell culture supernatant (Figure 1B). In addition to HSP70, we also detected the presence of mammalian ortholog of tick CD63 (exosomal marker, 30–65 kDa; endogenous-glycosylated) in both *Am* saliva and salivary gland-derived exosomal fractions (Figure 1B). Similarly, CD63 ortholog was also detected in *Is* salivary gland and ISE6 cell-derived exosomal fractions (Figure 1B). CD63 protein loads in *Am* salivary gland whole extracts (20 μg) are shown along with loads in ISE6 whole cells (20 μg) or cell-derived exosomal lysates (20 μl) (Supplementary Figure S3A). Both salivary gland and ISE6 whole cell lysates showed endogenous CD63 protein in addition to the glycosylated form, whereas in ISE6 cell-derived exosomal lysates, we detected only the heavily glycosylated form of CD63 (Supplementary Figure S3A). Gel image showing total protein profile serves as a loading control for each group of sample analyzed (Figure 1B and Supplementary Figure S3A). Some of the additional bands observed in total protein profile gel images from Figure 1B (indicated with asterisk) are highly enriched proteins in exosomal fractions that have been in the process of identification. The abundant levels of both HSP70 and CD63 tick orthologs suggest the presence of *in vivo* exosomes in tick saliva and salivary gland samples, in addition to the ISE6 cell-derived exosomes. Furthermore, combing of *Is* genome (not completely annotated) for CD63 ortholog sequences revealed three different CD63-like proteins with the following accession numbers *Is*CW001785, *Is*CW014150, and *Is*CW010731. PCR amplifications of CD63 showed products of 245, 261, and 241 bp, respectively, from both *Is* unfed females or ISE6 cells (Supplementary Figure S3B). ClustalW alignments of these three *Is* CD63-like protein amino acid sequences revealed 23–30% identity with sequences from *Aedes aegypti* mosquito protein, Tsp29Fb (Vora et al., 2018), mouse, or human orthologs (Supplementary Figures S4A,B). Phylogenetic analysis showed that two of the *Is* CD63-like proteins (*Is*CW001785 and *Is*CW014150) shared the same clade; however, the third one (*Is*CW010731) formed a divergent clade (Supplementary Figures S4B,C).

### Exosomes Derived From Tick Saliva, Salivary Glands, or ISE6 Cells Delay Migration and Tissue Repair of HaCaT Cells

**FIGURE 2:**
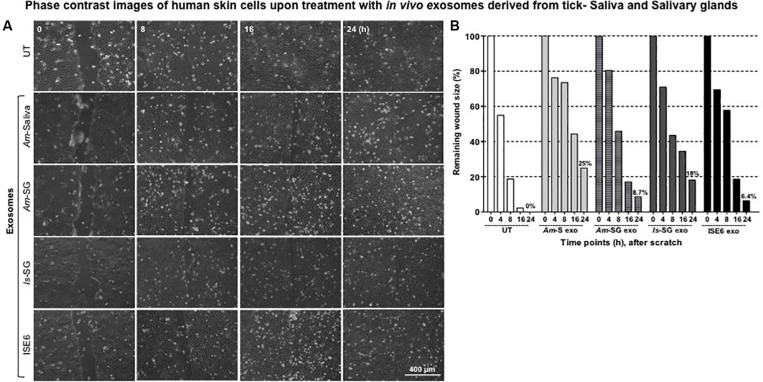
Exosomes derived from tick saliva, salivary glands, or ISE6 cells delay cell migration, closure, and repair of the scratch. **(A)** Phase contrast images of HaCaT cell monolayers treated with 20 μl of exosomal-pooled fractions (1–6) from either *Am* saliva, salivary glands, or *Is* salivary glands or ISE6 cells for 24 h. Images were collected immediately after tick exosome treatment that is considered as 0 h time point. Representative images are shown for each selected time points (of 0, 8, 16, and 24 h). HaCaT cell monolayers maintained as untreated (UT) group serves as the control. Images were obtained using 10 × magnification. Scale bar indicates 400 μm for each image per group/time point. **(B)** Quantitative measurement of remaining wound size percentages obtained from ImageJ at different time points of (0, 8, 16, and 24 h) posttreatment of *Am* saliva or salivary gland or *Is* salivary gland or ISE6 cell-derived exosomes is shown. *Am* represents *Amblyomma maculatum*, *Is* indicates *Ixodes scapularis*, and ISE6 denotes *Is*-derived cell line. Wounds at 0 h were considered as 100% for all groups, including the UT control. Percentages for remaining wound sizes at 24 h posttreatment of *in vivo* or *in vitro* tick exosomes are shown as bar graphs for comparison. Two-way ANOVA for significance is shown in Supplementary Table S1A.

To our knowledge, this is the first report about the discovery of *in vivo* exosomes in tick saliva or salivary glands. Next, we addressed the functional role or physiological significance of these tick exosomes at the tick–host skin interface. We found that HaCaT cells treated (for 24 h) with 20 μl of exosomal-pooled fractions (1–6) from groups of either *Am* saliva or salivary glands or *Is* salivary glands or ISE6 cells showed delays in cell migration and wound repair (noticeable at 16 and 24 h posttreatment) in comparison to the UT control group (Figure 2A and Supplementary Figure S5). Moreover, HaCaT cells treated with *in vivo* exosomes from tick saliva or salivary glands (from both *Am* or *Is*) had a much clear delay in cell migration and wound repair at 16 h in comparison to the UT control group (Figure 2A and Supplementary Figure S5). At 16 h, HaCaT cells treated with exosomes from ISE6 cells showed less delay and better wound closure in comparison to the *in vivo* tick exosomes (Figure 2A and Supplementary Figure S5). UT monolayers of HaCaT cells were considered as internal controls and are shown for comparison (Figure 2A and Supplementary Figure S5). For clear appearance of HaCaT cell monolayers, images from all time points (of 0, 4, 8, 16, and 24 h and including before scratches) and from all groups of exosome are shown (Supplementary Figure S5). Measurement of percentages for remaining wound sizes collected from images at different time points (of 0, 4, 8, 16, and 24 h) are shown in Figure 2B. At 24 h post wound scratch and treatment with tick exosomes (for 24 h), the percentages remaining of the wound sizes are in the following order from high to low: 25% (*Am* saliva), 18% (*Is* salivary gland), 8.7% (*Am* salivary gland), 6.4% (ISE6 cells), or 0% (UT control) (Figure 2B). All four groups of exosomes derived from *Am* saliva or salivary glands or *Is* salivary glands or ISE6 cells had a significant (*P* < 0.05) delay in wound closure when compared to the UT control group (Supplementary Table S1A). These data show that tick exosomes from saliva or salivary glands severely delayed wound healing that perhaps correlates to severe damage at the tick bite site.

### Cytokine Profiling From HaCaT Cells Revealed Differential Immune Responses by Tick Saliva, Salivary Glands, or ISE6 Cell-Derived Exosomes

**FIGURE 3:**
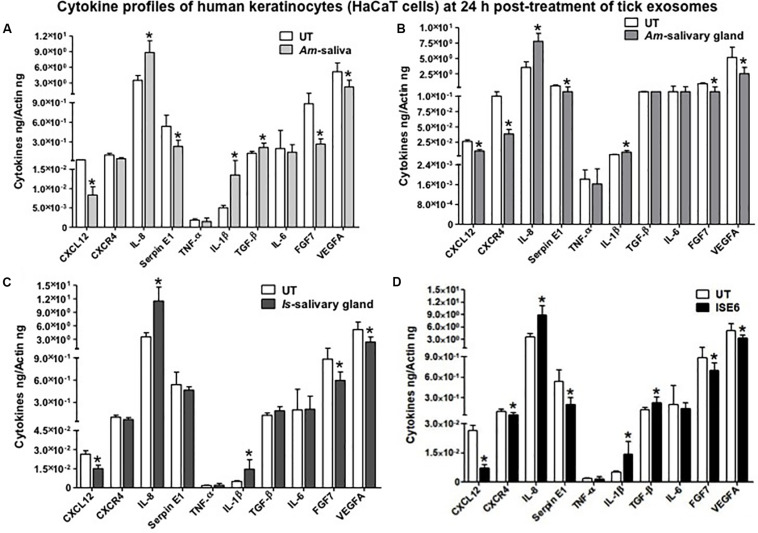
Tick exosome differentially regulates human skin cytokines and chemokines. Quantitative real-time PCR (QRT-PCR) analysis showing differential gene expressions of several cytokines and chemokines [CXCL12, CXCR4, IL-8, Serpin E1, tumor necrosis factor (TNF)-α, IL-1β, transforming growth factor (TGF)-β, IL-6, fibroblast growth factor (FGF)-7, and vascular endothelial growth factor A (VEGFA)] from HaCaT cells upon treatment with *in vivo* or *in vitro* tick exosomes derived from *Am* saliva **(A)** or salivary glands **(B)** or *Is* salivary glands **(C)** or ISE6 cells **(D)** for 24 h posttreatment. Untreated (UT) controls were used for each group of respective tick exosomes. Cytokine and chemokine transcript levels were normalized to human beta-actin. *Am* represents *Amblyomma maculatum*, *Is* indicates *Ixodes scapularis*, and ISE6 denotes *Is*-derived cell line. Asterisk indicates significance (*P* < 0.05) in comparison to the respective UT controls. *P*-value (*P* < 0.05) determined by Student’s two-tailed *t*-test is shown.

After 24 or 72 h posttreatment of HaCaT cells with 20 μl of exosomal-pooled fractions (1–6) from *Am* saliva or salivary glands or *Is* salivary glands or ISE6 cells, we analyzed the expression of several common cytokines or the ones that are highly expressed in human skin. QRT-PCR analysis revealed that some of the human cytokines were differentially regulated at both 24 and 72 h posttreatment with tick exosomes (Figures 3A–D and Supplementary Figures S6A–D). We found that at both 24 and 72 h time points, transcript levels of IL-8 (IL-8 or CXCL8) were significantly (*P* < 0.05) upregulated with the treatment of tick exosomes (from all four groups) in comparison to their respective UT controls (Figures 3A–D and Supplementary Figures S6A–D). Exosomes derived from *Am* saliva or salivary glands or *Is* salivary glands or ISE6 cells showed very similar and consistent induction of IL-8 (Figures 3A–D and Supplementary Figures S6A–D). In contrast to IL-8, we found that at both 24- and 72-h time points, SDF-1, also known as CXCL12, transcripts were significantly (*P* < 0.05) downregulated upon treatment with all four groups of tick exosomes in comparison to their respective UT controls (Figures 3A–D and Supplementary Figures S6A–D).

**FIGURE 4:**
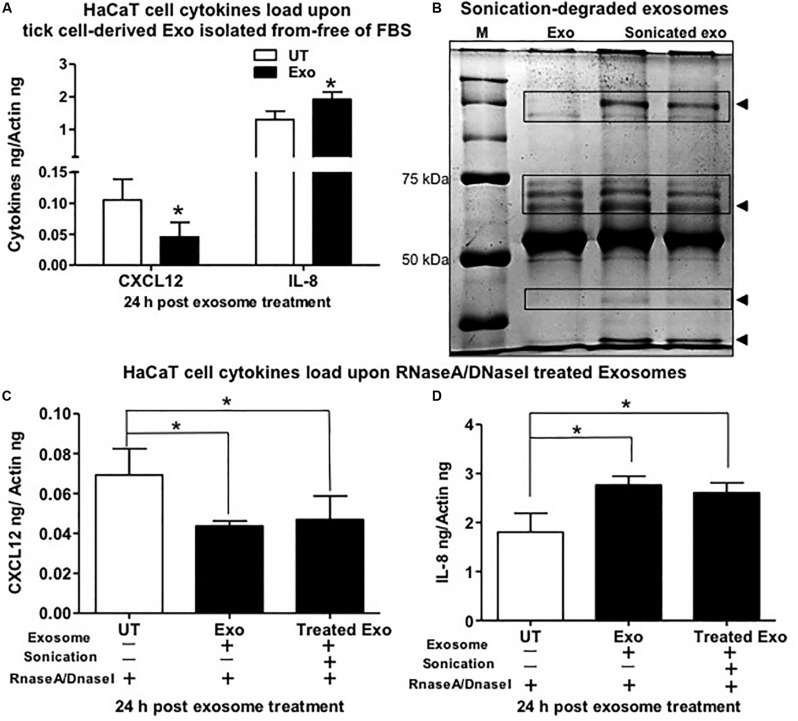
Tick exosomal proteins stimulate the regulation of interleukin-8 (IL-8) and C-X-C motif chemokine ligand 12 (CXCL12) and independent of bovine exosomes. **(A)** Quantitative real-time PCR (QRT-PCR) analysis showing gene expression of CXCL12 and IL-8 upon treatment (for 24 h) of HaCaT cells with exosomes collected from ISE6 cells cultured in Exo-free fetal bovine serum (FBS) medium (without any bovine exosomes). Black bar indicates HaCaT cells treated with tick cell-derived exosomes, and white bars indicate the untreated (UT) group. **(B)** Native polyacrylamide gel electrophoresis (PAGE) gel image showing degradation of tick exosomal proteins upon sonication. Sonicated exosome group is shown in duplicate with two lanes. Exo indicates exosomes that were not sonicated and serves as the control. M indicates protein ladder showing bands of 50 and 70 kDa. Arrowheads indicate the degraded proteins in sonicated exosome group in comparison to the not sonicated group of exosomes. QRT-PCR analysis showing CXCL12 **(C)** or IL-8 **(D)** gene expression in HaCaT cells treated with tick cell-derived exosomes processed for sonication followed by treatment with the mixture of RNaseA/DNaseI (last bar in each graph). HaCaT cells incubated with tick cell exosomes (not processed for sonication but treated with the mixture of RNaseA/DNaseI) serve as the control. HaCaT cells incubated with phosphate buffered saline (PBS) alone that is treated with the mixture of RNaseA/DNaseI serve as the internal control. Transcript levels of *cxcl12* and *Il-8* were normalized to human beta-actin. Asterisk indicates significance (*P* < 0.05) in comparison to respective UT controls. *P*-value determined by Student’s two-tailed *t*-test is shown.

The human keratinocyte growth factor or fibroblast growth factor (FGF)-7 that is involved in wound healing was also significantly (*P* < 0.05) downregulated (at both 24 and 72 h) upon treatment with tick exosome from all four groups in comparison to their respective controls (Figures 3A–D and Supplementary Figures S6A–D). Similar to FGF-7, the vascular endothelial growth factor A (VEGFA), also known as vascular permeability factor (VPF) that stimulates the formation of new blood vessels, showed significant (*P* < 0.05) downregulation (at both 24 and 72 h) upon treatment with tick exosomes (from all four groups) in comparison to their respective controls (Figures 3A–D and Supplementary Figures S6A–D). Two of the other cytokines that showed consistent and significant (*P* < 0.05) differences with tick exosome treatments (at both 24 and 72 h) include IL-1β and Serpin E1 [also known as endothelial plasminogen activator inhibitor (PAI)] (Figures 3A–D and Supplementary Figures S6A–D). Tumor necrosis factor alpha (TNF-α) transcripts were significantly (*P* < 0.05) upregulated only at 72 h posttreatment with exosomes (Supplementary Figures S6A–D). No significant differences were observed with TNF-α transcript levels at 24 h posttreatment with tick exosomes (Figures 3A–D). At 24 h posttreatment, transforming growth factor beta (TGF-β) transcript levels were significantly upregulated upon treatment with exosomes from *Am* saliva and ISE6 cells but not with treatment of *Am* or *Is* salivary gland-derived exosomes (Figures 3A–D). However, at 72 h posttreatment, TGF-β transcripts were significantly (*P* < 0.05) upregulated only in *Am* saliva-exosome group (Supplementary Figures S6A–D) but not in other groups. The C-X-C chemokine receptor type 4 (CXCR-4, also known as CD184) transcript levels were significantly (*P* < 0.05) downregulated at 24 h posttreatment with *Am* salivary glands or ISE6 cell-derived exosomes and showed no significant differences with *Am* saliva or *Is* salivary gland-derived exosomes in comparison to their respective UT groups (Figures 3A–D). At 72 h, CXCR-4 transcripts were unaltered in all four groups of exosomes (Supplementary Figures S6A–D). IL-6 that acts as both a pro-inflammatory cytokine and an anti-inflammatory myokine (cytokine released by muscle cells; myocytes) was not influenced (at both 24 and 72 h) by treatments with tick exosomes (Figures 3A–D and Supplementary Figures S6A–D). Next, we addressed if ISE6 cell-derived exosomes collected from complete L15 medium containing 5% FBS are regulating the cytokine expression independent of bovine exosomes from FBS. We performed similar experiments where HaCaT cells were treated with ISE6 cell-derived exosomes collected from ISE6 cells maintained in exosome-depleted FBS media. The expression levels of both CXCL12 (downregulated) and IL-8 (upregulated) were very similarly modulated when HaCaT cells were treated (for 24 h posttreatment) with these ISE6 cell-derived exosomes that are depleted of bovine exosomes (Figure 4A).

Furthermore, we tested whether extracellular RNA or DNA acts as a stimulus to differentially regulate the cytokines in human skin cells treated with ISE6 cell-derived exosomes. Native PAGE gel analysis of pretreated exosomes for sonication (to lyse their lipid bilayer membranes) showed that it degraded the tick exosomal proteins in comparison to the UT group of exosomes (Figure 4B). HaCaT cells treated with ISE6 exosomes (sonicated or not processed for sonication but both groups incubated with RNaseA/DNaseI mixture) or with an internal control group of PBS alone (as tick exosomes were suspended in PBS) showed no significant differences in cytokine expression of CXCL12 (Figure 4C) or IL-8 (Figure 4D). Both groups of exosomes (not sonication or processed for sonication) showed a significant (*P* < 0.05) reduction of CXCL12 or induction of IL-8 when compared to the internal control group of PBS alone-treated HaCaT cells (Figures 4C,D). In addition, we also tested the mouse macrophages (RAW 264.7 cell line) treated with exosomes derived from *Am* saliva or salivary glands or ISE6 cells and analyzed the expression levels of cytokines such as IL-1β, CXCL12, and TGF-β. No differences were observed for both IL-1β and TGF-β upon treatment with ISE6 exosomes (Supplementary Figure S7A). CXCL12 expression in RAW cells showed a significant (*P* < 0.05) reduction with *Am* tick salivary gland-derived exosomes; however, other groups showed no significant differences in comparison to the respective UT control group (Supplementary Figure S7A). Data from profiling of human cytokines suggested a differential immune response to tick exosomes at the bite site.

### Exosomes Derived From Tick Saliva, Salivary Glands, or ISE6 Cells Consistently Upregulated Interleukin-8 and Downregulated C-X-C Motif Chemokine Ligand 12 Secreted Proteins From HaCaT Cells

**FIGURE 5:**
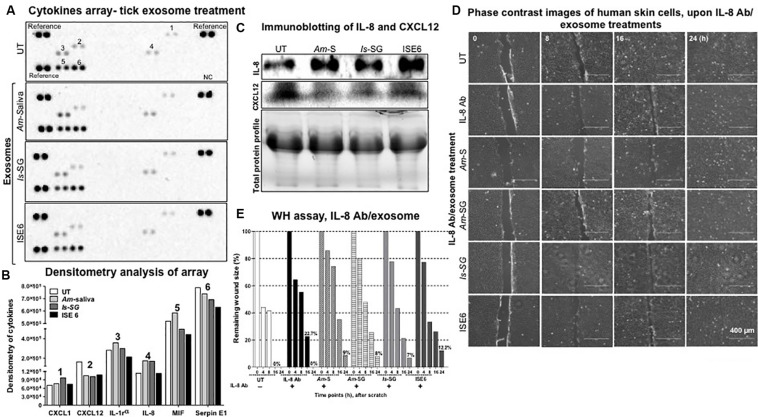
Tick exosome treatments showed consistent upregulation of interleukin-8 (IL-8) and downregulation of C-X-C motif chemokine ligand 12 (CXCL12) in HaCaT cells. **(A)** Multiple cytokine protein arrays with cell culture supernatants collected from HaCaT cells treated with *in vivo* or *in vitro* tick exosomes derived from *Am* saliva, or *Is* salivary glands, or ISE6 cells for 24 h posttreatment are shown. Images of nitrocellulose membranes with spotted molecule (as duplicates) are shown along with proteins spotted as a positive reference on three corners of each membrane. Proteins spotted as one negative control are shown at the bottom right and indicated as NC. Numbers indicated on membranes are related to CXCL1 (1), CXCL12 (2), IL-1 receptor (3), IL-8 (4), MIF (5), and Serpin E1 (6). Numbers indicate the molecules detected and evaluated for densitometry analysis. **(B)** Densitometry of spots detected on nitrocellulose membranes from multiple cytokine arrays of culture supernatants from HaCaT cells treated (for 24 h) with tick exosomes is shown. Different bar shades represent tick exosome-treated groups. **(C)** Immunoblotting analysis showing levels of secreted IL-8 and CXCL12 proteins in cell culture supernatants collected from HaCaT cells treated (for 24 h) with *in vivo* or *in vitro* tick exosomes derived from *Am* saliva, or *Is* salivary glands, or ISE6 cells. Total protein profiles are stain-free gel images that serve as the loading control. **(D)** Wound healing assay images from monolayers of HaCaT cells treated with IL-8 antibody (2 μg) for 12 h, followed by treatments (for 24 h) with tick exosomes from respective groups is shown. Phase contrast images collected at different time points (of 0, 8, 16, and 24 h) of post tick exosome treatments are shown. HaCaT cell wound closure images for IL-8 antibody alone or untreated (UT) groups collected at indicated time points serve as controls. Scale bar indicates 400 μm. **(E)** Quantitative measurement of remaining wound size percentages (obtained from ImageJ) from HaCaT cell monolayers treated with IL-8 antibody for 12 h, followed by treatment with tick exosomes at different time points (of 0, 8, 16, 24 h) is shown. *Am* represents *Amblyomma maculatum*, *Is* indicates *Ixodes scapularis*, and ISE6 denotes *Is*-derived cell line. Wounds at 0 h were considered as 100% for all groups, including IL-8 antibody alone or UT controls. Percentages for remaining wound sizes at 16 or 24 h posttreatment with *in vivo* or *in vitro* tick exosomes are shown as bar graphs for comparison. The –/+ indicates the presence or absence of IL-8 antibody. UT represents the untreated group that serves as the control in each respective experiment. Two-way ANOVA is shown in Supplementary Table S1B. WH indicates wound healing.

Both IL-8 and CXCL12 transcript loads were significantly (*P* < 0.05) modulated upon treatment with tick exosomes at 24 and 72 h posttreatment (Figures 3A–D and Supplementary Figures S6A–D). Next, we analyzed if these extracellular signaling molecules are also differentially secreted as proteins after tick exosome treatments by using a human cytokine proteome profiler array panel. Layout and Appendix table for human cytokine assay coordinates are provided in Supplementary Figures S7B,C. We found six secreted proteins to be differentially regulated in this profiler array. Interestingly, both IL-8 (increased) and CXCL12 (reduced) proteins were consistently regulated in cell supernatants collected from HaCaT cells treated (for 24 h) with *Am* saliva, *Is* salivary glands, or ISE6 cell-derived exosomes when compared to the UT control group (Figure 5A). We also noticed the presence of Serpin E1 to be consistently downregulated upon treatment with tick exosomes in comparison to the UT control (Figure 5A). The other three molecules modulated in HaCaT cell supernatants upon treatment with tick exosomes are CXCL1, macrophage migration inhibitory factor (MIF), and IL-1 receptor antagonist (IL-1RA) in comparison to the UT control (Figure 5A). Cell culture supernatants collected from UT HaCaT cells served as a control (Figure 5A). Densitometry analysis showed a differential regulation of IL-8, CXCL12, and other proteins in comparison to the UT control (Figure 5B). Furthermore, independent immunoblotting analysis performed with cell culture supernatants collected from HaCaT cells treated with tick exosomes and respective antibodies showed that secreted levels of IL-8 protein are dramatically upregulated and CXCL12 protein was considerably reduced in comparison to the UT control group (Figure 5C). Total protein profile gel image serves as a loading control (Figure 5C). Densitometry analysis for these immunoblots showed increased or decreased levels of IL-8 or CXCL12 proteins, respectively (Supplementary Figure 7D). Consistent with immunoblotting analysis, ELISA assay showed upregulation of secreted IL-8 protein in culture supernatants collected from HaCaT cells treated with exosomes derived from *Am* saliva or salivary glands or ISE6 cells in comparison to the UT control (Supplementary Figure 7E). These data suggest that tick exosomes affect the secretion of extracellular signaling molecules such as IL-8 and CXCL12 from human skin cells.

### Blocking of Interleukin-8 and C-X-C Motif Chemokine Ligand 12 Increased Tick Exosome-Mediated Effects of Delayed Cell Migration and Repair Process

In order to understand the importance of tick exosome-mediated IL-8 induction and CXCL12 reduction, we performed wound-healing assays (as described in the section “Materials and Methods”) in HaCaT cell monolayers. We found that HaCaT cell monolayers treated with IL-8 antibody in combination with tick exosome treatment (16 h) showed similar results as noted before (Figures 2A,B) with posttreatment of tick exosomes alone (Figure 5D and Supplementary Figure S8). At 16 h, we observed that wounds were neither closed in IL-8 antibody alone or IL-8 antibody treatment in combination with tick exosomes (Figure 5D and Supplementary Figure S8). However, we found that at 24 h, the group treated with IL-8 antibody alone was capable of closing the wound gap in comparison to the IL-8 antibody and tick exosomes combined groups (Figure 5D and Supplementary Figure S8). Similar observations were noted upon measurement of remaining wound size percentages at different time points (Figure 5E). At 24 h post wound scratch and treatment of exosomes (from *Am* saliva or salivary glands or *Is* salivary glands or ISE6 cells), the remaining wound sizes were calculated as 9, 8, 7, or 12.2%, whereas IL-8 antibody alone showed 0% wound remaining size, respectively (Figure 5E). No remaining wound size was seen in UT control group at 24 h, suggesting a complete wound closure in the absence of tick exosomes (Figure 5E). These data suggest that tick exosomes delay wound healing *via* an exaggerated immune response triggered by IL-8. Inhibition of IL-8 alone (*via* antibody blocking) showed delayed wound closure (at 16 h) in comparison to the UT control (Figure 5D and Supplementary Figure S8). In order to understand the significant role of IL-8 upon treatment with tick exosomes, we applied a genetic approach by using siRNA studies. Wound healing assays performed with HaCaT cells transfected with IL-8 siRNA for 24 h followed by treatment (24 h) with ISE6 cell-derived exosomes showed that silencing of IL-8 had no differences in comparison to the scrambled siRNA or the UT internal controls (Supplementary Figure S9A). At 24 h, all groups closed the scratch area without any differences (Supplementary Figure S9A). Measurement of remaining wound size percentages at 8 h showed no differences in wound closure with silencing of IL-8 siRNA (17%) when compared to the scrambled (34%) or UT control (30%) groups (Supplementary Figure S9B). Also, at 24 h, no significant differences were observed between IL-8 siRNA group or scrambled/UT controls (Supplementary Figure S9B and Supplementary Table S2A). The DNA agarose gel image shows the lower amounts of IL-8 transcripts in HaCaT cells in comparison to the scrambled or UT control groups (Supplementary Figure S9C). No differences were observed with actin transcript levels that serve as internal control (Supplementary Figure S9C). Densitometry analysis of the DNA agarose gel revealed lower levels of IL-8 transcripts in HaCaT cells transfected with siRNA in comparison to the scrambled/UT control groups (Supplementary Figure S9D).

**FIGURE 6:**
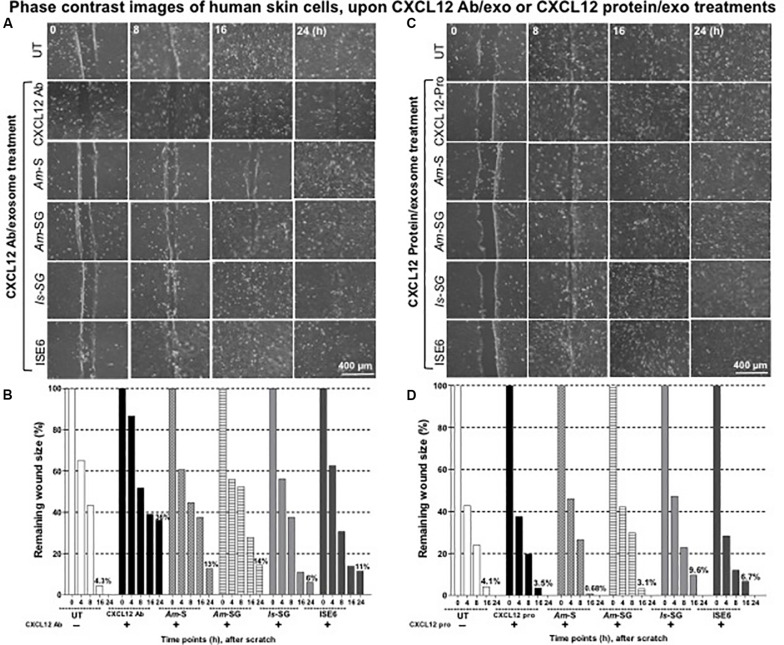
Blocking of C-X-C motif chemokine ligand 12 (CXCL12) *via* antibody increased but exogenous CXCL12 protein treatment restored tick exosome-mediated delayed wound healing and the repair process. Wound healing assay performed on monolayers of HaCaT cells treated with CXCL12 antibody (2 μg) for 12 h **(A)** or with CXCL12 purified protein (2 μg) for 4 h **(C)**, followed by treatments with tick exosomes derived from *Am* saliva or salivary glands, or *Is* salivary glands, or ISE6 cells is shown. Phase contrast images collected from different time points (0, 8, 16, and 24 h) of post tick exosome treatments are shown. HaCaT cell wound closure images for CXCL12 antibody alone or CXCL12 purified protein alone or untreated (UT) groups serve as internal controls. Scale bar indicates 400 μm. Quantitative measurement of remaining wound size percentages (obtained from ImageJ) of HaCaT cell monolayers treated with either CXCL12 antibody for 12 h **(B)** or with purified human CXCL12 protein for 4 h **(D)** followed by treatment with tick saliva, salivary glands, or ISE6 cell-derived exosomes at different time points (of 0, 8, 16, 24 h) is shown. *Am* represents *Amblyomma maculatum*, *Is* indicates *Ixodes scapularis*, and ISE6 denotes *Is*-derived cell line. Wounds at 0 h were considered as 100% for all groups, including CXCL12 antibody alone or CXCL12 protein alone or UT controls. Percentages for remaining wound sizes at 16 or 24 h posttreatment of tick exosomes are shown as bar graphs for comparison. The –/+ indicates the presence or absence of CXCL12 antibody or CXCL12 purified protein. Two-way ANOVA is shown in Supplementary Table S1B. Ab indicates antibody, and Exo denotes exosomes in the figure title.

In contrast to IL-8 antibody treatment, we found that CXCL12 antibody treatment completely failed to close wounds (even at 24 h posttreatment) either alone or when treated in mixture with tick exosomes (Figure 6A and Supplementary Figure S10). Noticeably, wider scratched areas were visualized (at 16 h posttreatment) upon treatment with CXCL12 antibody (alone) or in mixture with exosomes derived from *Am* saliva or salivary glands, *Is* salivary glands, or ISE6 cells (Figure 6A and Supplementary Figure S10). Images obtained from before scratch or immediately after tick exosome treatment (as 0 h) or continued until 24 h are shown for comparison (Supplementary Figures S8, S10). One group in each category treated with IL-8 or CXCL12 antibody alone served as an internal control in each respective independent experiment (Supplementary Figures S8, S10). Also, independent UT groups (without any treatments) were considered as internal controls in each antibody experiment (Supplementary Figures S8, S10). For clear appearance of cell monolayers and comparison with before scratch and inclusion of 4 h imaging group, we have shown enlarged images again for IL-8 and CXCL12 antibody alone treatments (Supplementary Figures S8, S10). Quantitative measurement analysis (at 24 h posttreatment) revealed wider areas of remaining wound sizes of about 36% for treatment with CXCL12 antibody alone, whereas CXCL12 antibody in combination with exosomes derived from *Am* saliva or salivary glands, *Is* salivary glands, or ISE6 cells showed 13, 14, 6, or 11%, respectively (Figure 6B). UT group showed (at 24-h treatments) 0% remaining wound size as expected (Figure 6B). In addition to IL-8 or CXCL12 antibody alone treatment controls, HaCaT cells were treated with IgG isotype control antibody. No differences in wound healing were observed with IgG antibody-treated group when compared to the UT control group at all tested time points (Supplementary Figure 11A). Also, the remaining wound size percentages showed no significant differences (Supplementary Figure 11B). No significant differences were also noted between the UT and IgG antibody-treated groups (Supplementary Table S2B). These data suggest that CXCL12 plays an important role in wound healing and the repair process and hence it is dramatically inhibited by tick exosomes to delay wound closures in skin.

### Exogenous Treatment of C-X-C Motif Chemokine Ligand 12 Protein Restored Cell Migration in Injured Areas Impaired by Exosomes From Tick Saliva, Salivary Glands, or ISE6 Cells

It was interesting to note that treatment with CXCL12 antibody alone showed wider remaining wound sizes and delayed wound healing either in the presence or absence of tick exosomes from all four groups. Next, we determined if treatment of HaCaT cells with human CXCL12 or SDF-1α (hBA-68) purified protein (2 μg) alone or in combination with tick *in vivo* or *in vitro* exosomes has any effect(s) in tick exosome-mediated delayed wound healing. It was noteworthy to find that treatment with 2 μg of human CXCL12 purified protein enhanced wound closure and the repair process at 16 h and in both cells treated with either CXCL12 protein alone or in combination with tick exosome groups (Figure 6C and Supplementary Figure S12). At 24 h posttreatment, we found that tick exosome (derived from *Am* saliva or salivary glands, *Is* salivary glands, or ISE6 cells)-mediated delayed wound closures/healings were completely restored in the presence of CXCL12 purified protein (Figure 6C and Supplementary Figure S12). These restored effects were comparable to the wound closures observed in CXCL12 protein alone-treated group or with the UT control group (Figure 6C and Supplementary Figure S12). The UT group, without any treatments, was considered as an internal control (Figure 6C and Supplementary Figure S12). Images collected before scratch and after immediate treatment (as 0 h) with respective groups of tick exosomes are shown as control groups (Supplementary Figure S12). For clear appearance of cell monolayers and comparison with before scratch and excluded 4-h time point group, enlarged images are shown for all panels in Supplementary Figure S12. Quantitative measurements of remaining wound sizes at 24 h showed 0% for all groups including CXCL12 protein alone or protein in combination with tick exosome treatments (Figure 6D). Measurements at 16 h post tick exosome treatment revealed that the CXCL12 protein restored wound sizes to be 0.68% (*Am* saliva exosomes), 3.1% (*Am* salivary gland-derived exosomes), 9.6% (*Is* salivary gland-derived exosomes), and 6.7% in ISE6 cell-derived exosomes in comparison to the UT group that showed 4.1% remaining wound size (Figure 6D). CXCL12 protein alone control group showed 3.5% wound remaining size (Figure 6D). In the presence of the CXCL12 protein, *Am* saliva-derived exosomes were considerably inhibited to impair wound closure and the repair process, and at 24 h, all groups closed the wounds (remaining wound size calculated as 0%) that were comparable to that of the UT group (Figure 6D). It is interesting to observe that in comparison to IL-8 or CXCL12 antibody treatment, the CXCL12 protein treatment showed significant (*P* < 0.05) restoration in wound closure with all four groups of tick exosomes in comparison to their respective mock controls. Significant (*P* < 0.05) differences between IL-8 and CXCL12 antibody-treated groups or CXCL12 protein-treated groups were noted by the two-way ANOVA (Supplementary Table S1B). In addition, wound healing assays performed with GST purified protein (2 μg) alone (control to the CXCL12-GST-tagged purified protein) or GST protein in combination with ISE6 cell-derived exosomes showed that GST protein alone had no effect, and it was comparable to that of the UT control group (Supplementary Figure S13A). However, GST protein in combination with ISE6 cell-derived exosomes showed delayed wound healing similar to the one noticed in Figure 2 and Supplementary Figure S13A. These data also showed that in contrast to CXCL12 protein, GST protein alone has no role in restoring tick exosome-mediated delays in wound repair (Supplementary Figure S13A). The quantitative measurements (at 24 h) showed 9.6% remaining wound sizes for GST protein in combination with ISE6 cell-derived exosomes, whereas GST protein alone or UT control groups showed 0% remaining wound sizes (Supplementary Figure S13B). At 16 h, no differences were seen for GST protein alone (11%) or UT groups (8.6%) (Supplementary Figure S13B). No significant differences were also noted between the UT and GST protein alone-treated groups (Supplementary Table S2C). Inhibition of both IL-8 and CXCL12 *via* antibodies in combination showed better delay in wound closures and the repair process (19%) in comparison to the UT control (Supplementary Figure S13B). These data suggest that enhanced expression and secretion of biologically active CXCL12 are very critical to suppress and restore the damaging effects of tick saliva or salivary gland-derived *in vivo* exosomes that impair wound healing and the repair process at the tick bite site.

## Discussion

To complete a successful blood meal, ticks evade the host defense system, including the very first barrier of skin keratinocytes and the responses from recruited immune cells at the bite site (Nuttall and Labuda, 2003, 2004; Leitner et al., 2011; Hanel et al., 2013; Bernard et al., 2014, 2015; Glatz et al., 2017; Zhou et al., 2018; Nuttall, 2019). The biological processes at the tick–host–virus interface have been well studied (Kazimirova et al., 2017). There are several important studies showing that saliva from ticks is enriched in molecules that modulate the host immune attack and tranquil the blood meal intake process (Bowman et al., 1997; Nuttall and Labuda, 2003, 2004; Brossard and Wikel, 2004; Valenzuela, 2004; Leitner et al., 2011; Bernard et al., 2014, 2015; Kotal et al., 2015; Scholl et al., 2016; Glatz et al., 2017; Vora et al., 2017; Wikel, 2018; Nuttall, 2019). In order to understand the transmission modes of tick-borne pathogens to human and other vertebrate hosts, we have previously shown that ISE6 cells secrete exosomes/EVs that mediate pathogen transmission to human skin keratinocytes and blood endothelial cells (Zhou et al., 2018). To our knowledge, this is the first report to implicate the tick saliva and salivary gland-derived *in vivo* exosomes in modulating the host immune response at the tick bite site to allow, ease, and mediate the blood-feeding process. Our discovery of the presence of exosomes in *Am* saliva or salivary glands or *Is* salivary glands suggests a functional role for tick salivary exosomes as extracellular signaling vesicles to coordinate and facilitate the process of tick feeding. Ticks have to elicit a variety of responses to modulate the sensing danger signals at the critical skin barrier (Bowman et al., 1997; Nuttall and Labuda, 2003, 2004; Brossard and Wikel, 2004; Valenzuela, 2004; Leitner et al., 2011; Bernard et al., 2014, 2015; Kotal et al., 2015; Scholl et al., 2016; Glatz et al., 2017; Vora et al., 2017; Wikel, 2018; Zhou et al., 2018; Nuttall, 2019). We assume that saliva molecules contained securely inside tick exosomes would be safe enough to avoid and shield themselves against these sensing dangers. This would allow ticks to win the first battle at the skin barrier that ultimately leads to successful blood feeding. Given that more force is required at this feeding point, salivary glands might continue to release exosomes with required cargo molecules (as shielded warriors) into the saliva pool and at the tick bite site. We have found that both tick saliva and salivary gland-derived exosomes were heterogeneous in the population with sizes from 30 to 200 nm in diameter (Figure 1A and Supplementary Figure S1A), suggesting differential cargo being transported *via* these EVs. The *in vivo* tick exosomes showed the presence of lipid bilayer membrane that maintains high stability for securely fusing the luminal cargo content into host keratinocytes. A higher expression of tick CD63 protein further confirmed that arthropod exosome membranes are highly stable with enriched tetraspanin domain containing glycoprotein decorations that perhaps allows interactions with host molecules. Our immunoblot data showing that CD63 is heavily glycosylated in tick cell-derived exosomes in comparison to the whole tick cells or tick salivary glands suggest that this molecule is highly modulated and perhaps transported in its readily functional or active form to maintain exosome membrane integrity and relay extracellular signaling. Also, the presence of three orthologs of CD63-like proteins in *Is* ticks further suggested the importance of these tetraspanins to play various/redundant roles during tick feeding. Our previous studies have shown abundant amounts of HSP70 as exosomal enriched protein in exosomes derived from cells of tick, mosquito, and mouse cortical neurons (Vora et al., 2018; Zhou et al., 2018, 2019). Detection of HSP70 in tick saliva and salivary gland-derived *in vivo* exosomes in addition to ISE6 cell-derived *in vitro* exosomes further confirmed a role for this chaperon at the tick bite site. We believe that the presence of HSP70 in tick exosomes plays a major role in blood feeding. Our independent study showed induction of this chaperon in ticks feeding on immunocompetent mice, whereas ticks feeding on immunodeficient animals had reduced HSP70 loads, perhaps due to less immune interference and disturbance (Vora et al., 2017). The presence and detection of CD63 and HSP70 suggest that cargo contents of tick exosomes derived from saliva and salivary glands are highly similar. Since ISE6 cells are a derivative of a mixture of salivary gland and midgut tissues, we assume that the cargo content from different tissues is perhaps similar or at least maintain important components such as tetraspanin glycoprotein, CD63. Given the pharmacological wonders of tick saliva, its complexity, chemical composition, and numerous functions (Bowman et al., 1997; Valenzuela, 2004; Kotal et al., 2015; Nuttall, 2019), we believe that exosomes are perhaps released with additional secreted proteins from saliva and hence would have more effects that could be synergic in mounting a higher immune response at the site of the tick bite. Also, in this study, we cannot compare the composition of exosomes from unfed or fed ticks and note that these differences could be based on the interspecific, stage-specific, or feeding-induced changes observed in ticks. An important study identified a novel plasminogen activator referred as Longistatin from the ixodid tick, *Haemaphysalis longicornis*, that was found to be resistant to the endothelial PAI-1 or Serpin E1 (Sugino et al., 2003). Serpins are serine protease inhibitors that mainly inhibit plasminogen activators and functions in fibrinolysis and blood clotting (Imamura et al., 2005; Prevot et al., 2006, 2009; Mulenga et al., 2009; Chalaire et al., 2011; Ibelli et al., 2014; Radulovic and Mulenga, 2017; Bakshi et al., 2018; Pongprayoon et al., 2019). Our current study showed that human Serpin E1 is consistently downregulated upon treatment with tick exosomes. Suppression of Serpin E1 in human keratinocytes upon treatment with tick exosomes suggests a tick response to allow tissue plasminogen activator or PLAT protein induction and breakdown of blood clots to allow continuous blood flow during tick feeding. Our current efforts are completely focused on understanding the detailed role of Serpin E1 during tick exosome-mediated blood feeding.

During tick bite, the saliva dribbles and suppresses blood clotting and the immune responses that reject these arthropod-mediated modulations (Bowman et al., 1997; Brossard and Wikel, 2004; Nuttall and Labuda, 2004; Valenzuela, 2004; Leitner et al., 2011; Kotal et al., 2015; Lewis et al., 2015; Glatz et al., 2017; Vora et al., 2017; Nuttall, 2019). It has been shown that blood plasma EV fibrinogen induces autoimmune-mediated spontaneous relapsing disease in a murine model of multiple sclerosis (Willis et al., 2019). Our previous work with HSP70 has shown that ticks elicit variable levels of fibrinogenolytic activities depending on the host immune background (Vora et al., 2017). Our present study explored the role of tick saliva, salivary glands, and ISE6 cell-derived exosomes in wound healing and the repair process in human keratinocytes. It was highlighting to find that tick exosomes delayed wound closure and the repair process, suggesting their role in mediating inhibition of tissue repair and facilitation of blood feeding at the bite site. Similar effects displayed by both *in vivo* and *in vitro* tick exosomes further confirmed their role in contributing to the delayed repair process in order to perhaps ease and facilitate blood feeding. Furthermore, consistent upregulation of IL-8 and repression of CXCL12 in HaCaT monolayers treated (24 and 72 h) with tick exosomes from respective groups suggested an important role for these molecules as regulators in wound healing and the repair process. Both IL-8 and CXCL12 have been suggested to play key roles during wound healing and the skin tissue repair process (Kamata et al., 2011; Ogawa et al., 2011; Jiang et al., 2012; Bollag and Hill, 2013; Scholl et al., 2016). IL-8 (CXCL8), a chemokine and a pro-inflammatory mediator, abundantly expressed in skin keratinocytes allows recruitment of neutrophils at this line of defense (Kamata et al., 2011; Jiang et al., 2012; Scholl et al., 2016). Several host cytokines including IL-8 have been modulated by tick salivary gland extracts (Hajnicka et al., 2005). Also, reduced IL-8 activity in context to several ixodid tick species (*Dermacentor reticulatus*, *Amblyomma variegatum*, *Rhipicephalus appendiculatus*, *Haemaphysalis inermis*, and *Ixodes ricinus*) was observed (Hajnicka et al., 2001). IL-8 has been shown to play important roles during the wound healing process. Polymorphonuclear leukocytes (PMNs) in human blisters and skin grafts donor site wounds showed a potential role for IL-8 as a chemoattractant (Rennekampff et al., 2000). In human keratinocyte assays, IL-8 recombinant protein (1 μg/ml) increased migration of these cells, and endogenous IL-8 played sequential functions in all phases of the human wound healing process (Rennekampff et al., 2000). In addition, autocrine/paracrine secretion of IL-8 supported the poly I:C-mediated HaCaT cell migration in scratch assays (Takada et al., 2017). An important study showed that tick saliva enhanced the production of pro-inflammatory mediators including IL-8 and IL-6 in dermal fibroblast cells (Scholl et al., 2016), whereas saliva when incubated on monocytes cocultured with *Borrelia burgdorferi* showed suppressive effects on the expression of IL-8 (Scholl et al., 2016). Our studies are in agreement with these findings that tick saliva, salivary gland, and ISE6 cell-derived exosomes induce IL-8 production and release from human keratinocytes. IL-8 gene transcripts and secreted protein were both upregulated, suggesting a magnified role for IL-8 in wound healing upon treatment with tick exosomes. We assume that induction of IL-8 is a host-mediated response, as inhibition of IL-8 (*via* antibody blocking, at 16 h) failed to close wounds in comparison to UT controls. At 24 h, IL-8 blocking showed wound closure in comparison to the IL-8 antibody and tick exosome combination treatment groups. We believe that upon IL-8 inhibition, CXCL12 or other molecules may play enhanced roles to repair the wounds. We did not find any changes with IL-6 upon treatment with tick exosomes at both 24 and 72 h, but we cannot exclude the possibility that in the absence of IL-8, IL-6 may play a compensatory role in the wound healing process. Upregulation of TNF-α levels at 72 h posttreatment but not at 24 h posttreatment with tick exosomes suggests its role in enhanced inflammation and exaggerated necrosis at the tick bite site during later stages of feeding. Upregulation of IL-1β upon treatment with tick exosomes suggests a local immune response to bite and salivary components. Early tick bite skin lesions at 24 h of *I. ricinus* attachments from 22 biopsy samples have shown elevated mRNA levels of pro-inflammatory cytokine IL-1β from macrophages and chemoattractant IL-8 (CXCL8) from neutrophils (Glatz et al., 2017). It has been assumed that after 24 h of tick attachment period, the primary inflammatory response weakens and an adaptive immune response arises and develops (Wikel, 1996a,b; Hanel et al., 2013; Glatz et al., 2017; Han et al., 2017). We have noted that upon tick exosome (both *in vivo* and *in vitro*) treatments, skin keratinocytes had increased levels of both IL-1β and IL-8 at 72 h posttreatment, but much higher levels were observed for both these transcripts at 24 h, an early period with tick exosome treatment. Other than IL-8 and IL-1β, we have observed differences with other cytokines such as TGF-β that show upregulation with treatment of tick saliva or ISE6 cell-derived exosomes. It has also been reported that tick salivary gland extract binds to growth factors such as TGF-β1, platelet-derived growth factor (PDGF), FGF-2, and hepatocyte growth factor (HGF) (Hajnicka et al., 2011). We have noted the reduction of FGF-7 and VEGFA cytokines. The significance for this differential expression will be addressed in our future studies. We assume that tick exosomes may either lyse up to release the luminal content or perhaps fuse with keratinocytes and other recruited immune cells such as neutrophils, macrophages, and dendritic cells. Several studies have examined the role of tick saliva on monocytes, macrophages, dendritic, and several other immune cells (Nuttall and Labuda, 2004; Valenzuela, 2004; Prevot et al., 2006; Kotal et al., 2015; Scholl et al., 2016; Radulovic and Mulenga, 2017; Nuttall, 2019). No or inconsistent differences found in expression levels of IL-1β, CXCL12, and TGF-β from mouse macrophages suggest that tick exosomes modulate these molecules in human skin barrier keratinocytes specifically and perhaps differentially in other immune cells. We cannot rule out the effect of incubation of tick exosomes with macrophages and analyze the expression of cytokines and chemokines at the later time points such as 72 h post infection. We are in the process of exploring the role of tick saliva and salivary gland-derived exosomes on various other immune cells and their responses in the production of cytokines and chemokines.

**FIGURE 7:**
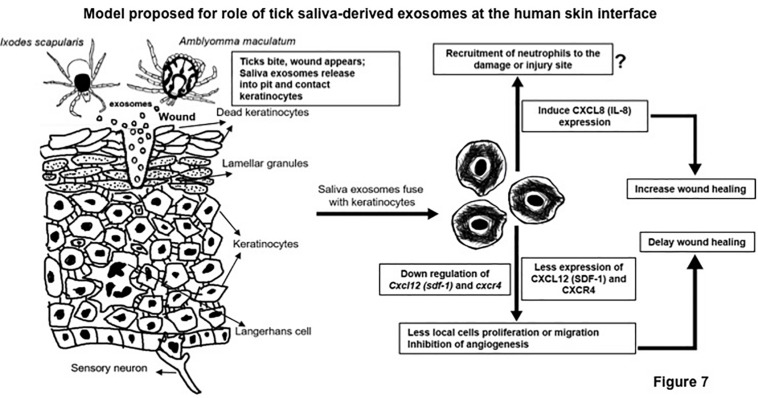
Tick saliva exosomes modulate human skin interface. Model showing tick bite site or hypostome injected into a damaged skin pit, with saliva deposit or spit from *Am* or *Is* ticks. We propose that the release of *in vivo* exosomes into the saliva at the wound/bite site exaggerates the residential keratinocytes and perhaps residential Langerhans cells to modulate cytokines or chemokines such as interleukin-8 (IL-8) and/or C-X-C motif chemokine ligand 12 (CXCL12) to control tissue damage, injury, wound healing, and repair processes. This may allow, ease, and promote successful completion of tick blood feeding. *Am* represents *Amblyomma maculatum*, and *Is* indicates *Ixodes scapularis*.

So far, there are no reports showing a role for CXCL12 in facilitating tick exosome-mediated immune response(s) during tick feeding or aiding the host defense at the tick bite site. CXCL12 has been shown to play sufficient roles during wound healing and the tissue repair process (Gillitzer, 1996; Gillitzer et al., 2000; Restivo et al., 2010; Feng et al., 2014; Kim et al., 2015; Vagesjo et al., 2018). Wound healing is a highly dynamic and regulated process with several subprocesses or phases. We considered the phases that required the integration of both biological and molecular events such as secretion of cytokines, cell migration, and repair of the scratch processes in this study. We believe that induction of IL-8 is a host-mediated independent event. Inhibition of IL-8 delayed wound healing and the repair process. However, in the presence of tick exosomes, skin cells further upregulated (perhaps three folds) this molecule to support healing and the repair process. We also believe that in the presence of IL-8 antibody, the inhibition is much enhanced as the secreted IL-8 protein is blocked efficiently (by the antibody binding), and the effects are stronger in comparison to the genetic approach by siRNA silencing of the IL-8 transcript. Proteomic analysis of whole human saliva-derived exosomes has shown the presence of HSP70, Alix, Tsg101, and CD26. The latter molecule has been shown to metabolically cleave chemokines such as CXCL11 and CXCL12 to regulate the local immune defense against oral microbes (Ogawa et al., 2011). Consistent suppression of CXCL12 upon treatment (at early 24 h or late 72 h) with *in vivo*- or *in vitro*-derived tick exosomes suggests a very critical role played by this molecule in host defense at the human skin. Our data clearly suggested that CXCL12 mRNA transcripts and secreted protein were severely inhibited by tick exosomes derived from either *in vivo* or *in vitro* group. Downregulation of CXCR4 (a receptor for CXCL12) at early 24 h posttreatment of tick exosomes further suggested the reduced uptake, production, secretion, and binding of CXCL12 ligand to its receptor (CXCR4). Inhibition of CXCL12 by antibody blocking in combination with tick exosome treatments especially from *Am* saliva or salivary glands showed a dramatic delay in wound closure and repair at both 16 and 24 h posttreatment. Importantly, restoration in wound healing and repair observed after treatment with CXCL12 purified protein clearly suggested a potential role for this molecule in the inhibition of inflammation, damage, and enhancement of wound closure and tissue repair at the tick bite site. It was surprising to note that only 2 μg of CXCL12 protein was sufficient to protect the skin keratinocytes and mediate wound closure and the repair process. Our proposed model (Figure 7) suggests that induction of CXCL12 and perhaps IL-8 in skin keratinocytes would support to ease inflammation, reduce skin damage, and promote cell proliferation, wound healing, and tissue repair at the tick bite site. Also, exogenous use of stable CXCL12 protein as lotion or spray at the bite site may inhibit the exaggerated responses triggered by tick exosomes. We assume that the use of CXCL12 as a potential therapeutic on human skin may affect tick saliva exosomes and/or prevent blood feeding by inducing the host defense to promote rapid wound healing and the repair process. Our future studies will target such applied research in *in vivo* mouse models. Overall, our study showed a novel role for *in vivo/in vitro* exosomes in facilitating tick blood feeding by modulating important host molecules such as IL-8 and CXCL12 from skin keratinocytes. This work also suggested the therapeutic potential of CXCL12 in preventing not just tick-related but also other skin inflammation, tissue damage, injury, skin burns, and wound closures. Taken together, this study opens new thoughts on how arthropod exosomes in saliva can take the toll to facilitate and allow tick feeding at the confrontation site and conquest a difficult and dangerous battle for that delicious blood meal.

## Significance Statement

Our work suggests that induction of CXCL12 and perhaps IL-8 in skin keratinocytes would ease inflammation, reduce skin damage, and promote cell proliferation, wound healing, and tissue repair at the tick bite site. Also, exogenous use of stable CXCL12 protein may inhibit the exaggerated responses triggered by exosomes upon tick bite. We assume that the use of CXCL12 as a potential therapeutic on human skin may affect tick saliva exosomes and/or prevent tick blood feeding by inducing the host defense to promote rapid wound healing and the repair process. Overall, our study showed a novel role for exosomes in facilitating tick blood feeding by modulating important host molecules such as IL-8 and CXCL12 from skin keratinocytes.

## Data Availability Statement

All datasets generated for this study are included in the article/Supplementary Material.

## Ethics Statement

The animal study was reviewed and approved by Institutional Animal Care and Use Committee (IACUC) at University of Southern Mississippi (protocol # 15101501).

## Author Contributions

WZ and HS performed all the experiments. WZ, GN, and HS discussed, analyzed, and interpreted the data in several settings. GN generated the salivary gland tissue samples from *Is* unfed adult female ticks and purified the GST protein from *E. coli*. FT and SK performed the partial blood feeding of adult female *Am* ticks on sheep, collected the saliva from independent batches, and generated the salivary gland tissues from *Am* ticks. MW and MS performed the cryo-electron microscopy (on two independent shipped samples), collected the imaging data, and performed the careful analysis of *in vivo* exosomes and sizes. Independently, JW also performed the cryo-EM (for two independent times) analysis and in addition performed TEM analysis/negative staining to reveal the presence of exosomes from *Am* tick saliva and salivary glands of both *Am* and *Is* ticks. All the authors read and edited the manuscript. HS collected all required materials and reagents, designed and coordinated the entire study, compiled and organized all the data, supervised the overall investigations, and wrote the manuscript.

## Conflict of Interest

The authors declare that the research was conducted in the absence of any commercial or financial relationships that could be construed as a potential conflict of interest.

## Supplementary Material

FIGURE S1 Cryo-EM imaging and measurement of *in vivo* exosomes derived from *Am* saliva and salivary glands. **(A)** Additional cryo-EM (electron microscopy) images collected from two independent institutions (University of Texas Medical Branch; UTMB and Indiana University; IU) showing exosomes isolated from *Am* saliva or salivary gland tissues. Scale bar indicates 100 nm (UTMB), or 200 or 500 nm (IU), respectively. For better comparison, scales have been provided close to individual exosomes. For images from IU on right side, insets show detailed structure of exosomes from the field. Measurement of diameters shows heterogeneous population of *in vivo* exosomes from saliva or salivary glands. Particle diameter measurement (in nm) analysis against the concentration of particles to determine the exosome sizes from *Am* saliva (indicated as *Am*-S) **(B)** or *Is* salivary gland-derived exosomes (represented as *Is*-SG) **(C)** is shown. N represents number of particles determined or counted using the TS-300 filter that measured 50–300 nm particle sizes. Scatter plot graphs on right in both (B) and (C) shows number of particles counted during the transit time (indicated as N) in Spectradyne nCS1 nanoparticle analyzer. *Am* represents (*Amblyomma maculatum*) and *Is* indicates (*Ixodes scapularis*) ticks.

FIGURE S2 Detection of CD63 from brain tissue-derived exosomal fractions and estimation of proteins from tick exosomes. **(A)** Immunoblotting analysis showing detection of exosomal enriched marker CD63 in six different exosomal fractions prepared from C57/BL6 wild type mice brain tissue. Total protein profile is shown from the stain free gel image that serves as loading control. **(B,C)** BCA measurement assay showing the estimated protein amounts at an optical density of 562 nm (shown in *Y*-axis) and concentration of tick exosomal proteins (in μg/μl). The R^2^ value is shown for the standards.

FIGURE S3 Enhanced CD63 levels in tick cell-derived exosomes and amplification of *Is* CD63-like molecules. **(A)** Immunoblotting analysis showing detection of exosomal enriched marker CD63 in *Am* whole salivary gland lysates, tick cell-derived exosomes, and whole tick cell lysates. Arrowheads indicate the endogenous (lower) and glycosylated (middle and upper) protein in salivary gland and ISE6 cell and exosomal lysates. Total protein profile is shown from the stain free gel image that serves as loading control. M indicate protein ladder and SG denotes salivary glands. **(B)** PCR amplification of CD63-like genes from *Is* unfed female ticks or ISE6 cells cDNA is shown. Three different fragments were amplified, and bands of approximately 245, 261 and 241 bp (denoted by arrowheads) were detected on 1% agarose gel for ISE6 cells. *Is* unfed female ticks showed amplified product for two CD63-like molecules (*Is*CW014150, and *Is*CW010731). NTC denotes no template control, and NTC is shown for respective primer pairs. M represents DNA ladder and *Is* shows *Ixodes scapularis*. Arrowhead indicates the 200 bp reference bands in DNA ladder.

FIGURE S4 Alignment, phylogenetic analysis and identity of *Is* CD63-like proteins. **(A)** Deduced *Is* CD63-like amino acid sequence alignments (with other orthologs) using ClustalW program in DNASTAR Lasergene is shown. Residues that match are shaded in black color. GenBank accession numbers for *M. musculus* and *H. sapiens* CD63 sequences are shown. VectorBase accession numbers for three of the *Is* CD63-like proteins, and *A. aegypti* Tsp29Fb are provided. Total length of the amino acid sequence is provided at left end of each sequence. **(B)** Phylogenetic analysis was performed in DNASTAR by ClustalW slow/accurate alignment method using Gonnet as default value for protein weight matrix. Scale at the bottom denotes amino acid substitutions per 100 amino acid residues. Bootstrap and seed numbers are provided. **(C)** Percent identity (horizontally above black boxed diagonal) and divergence (vertically below black boxed diagonal) of *Is* CD63-like nucleotide sequence in comparison to *A. aegypti* Tsp29Fb, *M. musculus* and *H. sapiens* CD63 sequences is shown.

FIGURE S5 Exosomes derived from tick saliva, salivary glands, or ISE6 cells delay wound closure and repair in skin keratinocytes. Phase contrast images of HaCaT cells monolayers treated with 20 μl of exosomal-pooled fractions (1–6) from either *Am* saliva or salivary glands or *Is* salivary glands or ISE6 cells for 24 h is shown. Images were obtained before any treatments and shown as before scratch. Scratch generated cell images before treatment with tick exosomes are shown as 0 h. Representative images are shown for each time points (of 0, 4, 8, 16, and 24 h) post tick exosome-treatments. Images from time points of 0, 8, 16, and 24 h are previously shown in Figure 2 and are repeated in this figure for better comparison with inclusion of before scratch and 4 h time point group. HaCaT cell monolayers maintained as untreated (UT) group serve as control. Images were obtained using EVOS FL system and 10X magnification. Scale bar indicates 400 μm for each image per group/time point. *Am* represents (*Amblyomma maculatum*), *Is* indicates (*Ixodes scapularis*) and ISE6 denotes *Is*-derived cell line. Two-way ANOVA analysis is shown in Supplementary Table S1A.

FIGURE S6 Human skin cells cytokine analysis revealed differential regulation by tick saliva, salivary gland, or ISE6 cell-derived exosomes. QRT-PCR analysis showing levels of different cytokines/chemokines (CXCL12, CXCR4, IL-8, Serpin E1, TNF-α, IL-1β, TGF-β, IL-6, FGF-7 and VEGFA) from HaCaT cells **(A–D)** treated (for 72 h) with exosomes derived from *Am* saliva or salivary glands, *Is* salivary glands or ISE6 cells. Transcript levels were compared to the levels of the cytokine or chemokine levels in untreated (UT) HaCaT cells. Cytokine levels were normalized to human beta-actin, respectively. Asterisk indicates significance (*P* < 0.05) in comparison to respective untreated controls. *P*-value determined by Student’s two-tailed *t*-test is shown.

FIGURE S7 Cytokine expression in mouse macrophages, protein array profile layouts and detection of IL-8 upregulation by ELISA. **(A)** QRT-PCR analysis showing expression of IL-1β, TGF-β and CXCL12 from mouse macrophages (RAW264.7 cell line) treated (for 24 h) with exosomes from *Am* saliva or salivary glands, or ISE6 cells. Each cytokine load is compared to its respective untreated control group. UT indicates untreated. Transcript levels in RAW 264.7 cells were normalized to mouse beta-actin, respectively. **(B)** Layout of human cytokine assay coordinates spotted on four of the nitrocellulose membranes purchased from R&D systems is shown. **(C)** Appendix table for assay coordinates showing the details of cytokines/chemokines spotted in duplicate on the nitrocellulose membrane is provided from the vendor’s website. The Reference proteins as positive control are spotted on membranes at A1, 2; E1, 2 and A19, 20 whereas E19, 20 were negative controls for the assay. **(D)** Densitometry analysis showing differences in secreted protein levels of IL-8 or CXCL12 in comparison to the untreated (UT) control. *Am* represents (*Amblyomma maculatum*), *Is* indicates (*Ixodes scapularis*) and ISE6 denotes *Is*-derived cell line. **(E)** ELISA assay showing IL-8 expression in cell culture supernatants collected from HaCaT cells treated (24 h) with exosomes from *Am* saliva or salivary glands, or ISE6 cells. SG indicates salivary glands. Asterisk indicates significance (*P* < 0.05) in comparison to respective untreated controls. *P*-value determined by Student’s two-tailed *t*-test is shown.

FIGURE S8 Blocking of IL-8 via antibody followed by treatment with exosomes derived from tick saliva, salivary glands, or ISE6 cells further delays wound closure and repair. Phase contrast images of HaCaT cell monolayers treated with IL-8 antibody (2 μg) for 12 h, followed by treatments with 20 μl of exosomal-pooled fractions (1-6) from either *Am* saliva, or salivary glands or *Is* salivary glands or ISE6 cells for 24 h is shown. Images of HaCaT cell monolayers collected before any treatments served as before scratch internal control. Scratches were generated and images collected immediately after scratches as 0 h, followed by treatment (for 24 h) of HaCaT cells with tick exosomes is shown. Representative images are shown for each time points (of 0, 4, 8, 16, and 24 h) post tick exosome-treatments. Images from time points of 0, 8, 16, and 24 h are previously shown in Figure 5D, and repeated in this figure for comparison with before scratch and 4 h group. HaCaT cell monolayers that were maintained as untreated (UT) served as control. Images were obtained using EVOS FL system and 10X magnification. Scale bar indicates 400 μm for each image/group/time point. *Am* represents (*Amblyomma maculatum*), *Is* indicates (*Ixodes scapularis*) and ISE6 denotes *Is*-derived cell line. Two-way ANOVA analysis is shown in Supplementary Table S1B.

FIGURE S9 Silencing of IL-8 via siRNA followed by treatment with exosomes derived from tick cells. **(A)** Phase contrast images of HaCaT cell monolayers treated with IL-8 siRNA (∼1 μg) for 24 h, followed by scratches and treatments with 20 μl of exosomal-pooled fractions (1–6) from ISE6 cells for 24 h is shown. Images were collected immediately after scratches as 0 h. Representative images are shown for each time points (of 0, 4, 8, 16, and 24 h) post tick exosome-treatments. Scrambled (Scr) siRNA group serve as control. HaCaT cell monolayers that were maintained as untreated (UT) served as internal control. Images were obtained using EVOS FL system and 10X magnification. Scale bar indicates 400 μm for each image/group/time point. **(B)** Quantitative measurement of percentages of remaining wound sizes at different time points of 0, 4, 8, 16, and 24 h are shown for IL-8 siRNA, or scrambled siRNA in combination with ISE6 cell-derived exosomes. Wounds at 0 h were considered as 100% for all groups, including IL-8 siRNA, or scrambled siRNA or untreated (UT) controls. Percentages for remaining wound sizes at 8 or 24 h posttreatment of ISE6 cell-derived exosomes are shown on bar graphs for comparison. Two-way ANOVA analysis is shown in Supplementary Table S2A. **(C)** PCR amplification and DNA agarose gel electrophoresis (1.2% gel) of HaCaT cells collected from wound healing assay showing transcripts of IL-8 in siRNA treated, scrambled, or untreated groups. The actin transcripts are shown as internal control. *M* indicate DNA ladder and arrowhead denotes an amplified product of 219 bp. **(D)** Densitometry analysis showing the reduction in IL-8 amplified product in siRNA treated group in comparison to the scrambled or untreated controls.

FIGURE S10 Blocking of CXCL12 followed by treatment with exosomes derived from tick saliva, salivary glands, or ISE6 cells further delays wound closure and repair. Phase contrast images of HaCaT cell monolayers treated with CXCL12 antibody (2 μg) for 12 h, followed by treatment with 20 μl of exosomal-pooled fractions (1–6) from either *Am* saliva, or salivary glands or *Is* salivary glands or ISE6 cells for 24 h is shown. Images of HaCaT cells before scratch or any treatments served as internal control group. Images collected immediately after scratches are considered as 0 h and shown for comparison. Scratches were generated and tick exosomes were treated for 24 h and representative images are shown for each time points (of 0, 4, 8, 16, and 24 h). Images from time points of 0, 8, 16, and 24 h are previously shown in Figure 6A, and repeated here for comparison with before scratch and 4 h time point groups. HaCaT cell monolayers that were maintained as untreated (UT) served as control group. Images were obtained using EVOS FL system and 10X magnification. Scale bar indicates 400 μm for each image per group/time point. *Am* represents (*Amblyomma maculatum*), *Is* indicates (*Ixodes scapularis*) and ISE6 denotes *Is*-derived cell line. Two-way ANOVA analysis is shown in Supplementary Table S1B.

FIGURE S11 Treatment of isotype control IgG antibody did not interfere with wound healing process. **(A)** Phase contrast images of HaCaT cell monolayers treated with isotype control IgG antibody (2 μg) for 24 h, followed by scratches and treatments with 20 μl of exosomal-pooled fractions (1–6) from ISE6 cells for 24 h is shown. Images were collected immediately after scratches as 0 h. Representative images are shown for each time points (of 0, 4, 8, 16, and 24 h) post tick exosome treatments. Untreated (UT) group served as internal control. Images were obtained using EVOS FL system and 10X magnification. Scale bar indicates 400 μm for each image/group/time point. **(B)** Quantitative measurement of percentages of remaining wound sizes at different time points of 0, 4, 8, 16, and 24 h are shown for IgG isotype antibody group treated with ISE6 cell-derived exosomes or untreated control. Wounds at 0 h were considered as 100% for all groups, including IgG isotype antibody group or untreated (UT) control. Percentages for remaining wound sizes at 16 or 24 h posttreatment of ISE6 cell-derived exosomes are shown on bar graphs for comparison. Two-way ANOVA analysis is shown in Supplementary Table S2B.

FIGURE S12 CXCL12 exogenous protein treatment restored delayed wound closure and repair. Images from purified GST-tagged CXCL12 protein (2 μg) treated on HaCaT cell monolayers for 4 h, then followed by treatments with 20 μl of exosomal-pooled fractions (1–6) from either *Am* saliva or salivary glands or *Is* salivary glands or ISE6 cells for 24 h are shown. Images of HaCaT cell monolayers before scratch served as internal control group. Images collected immediately after scratches and before treatment with tick exosomes are shown as 0 h. Representative phase contrast images are shown for each time points (of 0, 4, 8, 16, and 24 h) post tick exosome treatments. Images from time points of 0, 8, 16, and 24 h are previously shown in Figure 6C and repeated in this figure for comparison with before scratch and 4 h time point groups. HaCaT cell monolayers, that were maintained as untreated (UT) serve as control. Images were obtained using EVOS FL system and 10X magnification. Scale bar indicates 400 μm for each image per group/time point. *Am* represents (*Amblyomma maculatum*), *Is* indicates (*Ixodes scapularis*) and ISE6 denotes *Is*-derived cells. Two-way ANOVA analysis is shown in Supplementary Table S1B.

FIGURE S13 Treatment of GST purified protein does not influence tick exosome-mediated delay in wound closure and repair. **(A)** Purified GST protein alone (2 μg) treated on HaCaT cell monolayers for 4 h, followed by treatments with 20 μl of exosomal-pooled fractions (1–6) from ISE6 cells for 24 h is shown. Images from IL-8 and CXCL12 combination antibodies (2 μg, each) treatment is also shown as control. Images of HaCaT cell monolayers taken before scratch serve as control. Scratches were generated and images were collected immediately after scratches as 0 h. Representative phase contrast images are shown for each time points (of 0, 4, 8, 16, and 24 h) post tick exosome treatments. HaCaT cell monolayers, that were maintained as untreated (UT) serve as control. Images were obtained using EVOS FL system and 10X magnification. Scale bar indicates 400 μm for each image per group/time point. **(B)** Quantitative measurement of percentages of remaining wound sizes at different time points of 0, 4, 8, 16, and 24 h are shown for GST protein alone, or GST protein in combination with ISE6 cell-derived exosome treatment or treatments with combination of IL-8 and CXCL12 antibodies is shown. Wounds at 0 h were considered as 100% for all groups, including GST protein alone or untreated (UT) controls. Percentages for remaining wound sizes at 16 or 24 h posttreatment of ISE6 cell-derived exosomes are shown on bar graphs for comparison. Two-way ANOVA analysis is shown in Supplementary Table S2C.

TABLE S1 Two-way ANOVA analysis for HaCaT cells incubated with ISE6 cell-derived exosome treatments and treatments with IL-8/CXCL12 Antibodies or CXCL12 protein. **(A)** Statistical significance is indicated as Yes or No below black-boxed diagonal and *P-*value for each comparison is indicated above black boxed diagonal. All significant and marginal significant values are in bold text. (B) Statistical significance was compared to mock-treated group. Time point posttreatment is shown. All significant and marginal significant values are in bold text.

TABLE S2 Two-way ANOVA for HaCaT cells incubated with ISE6 cell-derived exosome and treatments with IL-8 siRNA and isotype control IgG antibody. In all panels, Source of variations, SS, df, MS, F, P value, and *F* crit values are shown. *P* > 0.05 is not considered significant.

TABLE S3 Oligonucleotides used in this study.

## References

[B1] Bakshi M. Kim T. K. Mulenga A. (2018). Disruption of blood meal-responsive serpins prevents *Ixodes scapularis* from feeding to repletion. *Ticks Tick Borne Dis.* 9 506–518. 10.1016/j.ttbdis.2018.01.001 29396196 PMC5857477

[B2] Bernard Q. Gallo R. L. Jaulhac B. Nakatsuji T. Luft B. Yang X. (2016). Ixodes tick saliva suppresses the keratinocyte cytokine response to TLR2/TLR3 ligands during early exposure to Lyme borreliosis. *Exp Dermatol.* 25 26–31. 10.1111/exd.12853 26307945

[B3] Bernard Q. Jaulhac B. Boulanger N. (2014). Smuggling across the border: how arthropod-borne pathogens evade and exploit the host defense system of the skin. *J. Invest. Dermatol.* 134 1211–1219. 10.1038/jid.2014.36 24552683

[B4] Bernard Q. Jaulhac B. Boulanger N. (2015). Skin and arthropods: an effective interaction used by pathogens in vector-borne diseases. *Eur. J. Dermatol.* 25(Suppl. 1) 18–22. 10.1684/ejd.2015.2550 26083670

[B5] Bollag W. B. Hill W. D. (2013). CXCR4 in epidermal keratinocytes: crosstalk within the skin. *J. Invest. Dermatol.* 133 2505–2508. 10.1038/jid.2013.271 24129780 PMC3966191

[B6] Bowman A. S. Coons L. B. Needham G. R. Sauer J. R. (1997). Tick saliva: recent advances and implications for vector competence. *Med. Vet. Entomol.* 11 277–285. 9330260 10.1111/j.1365-2915.1997.tb00407.x

[B7] Brossard M. Wikel S. K. (2004). Tick immunobiology. *Parasitol* 129(Suppl.) S161–S176. 10.1017/s0031182004004834 15940820

[B8] Carneiro J. R. Fuzii H. T. Kayser C. Alberto F. L. Soares F. A. Sato E. I. (2011). IL-2, IL-5, TNF-alpha and IFN-gamma mRNA expression in epidermal keratinocytes of systemic lupus erythematosus skin lesions. *Clinics* 66 77–82. 10.1590/s1807-59322011000100014 21437440 PMC3044589

[B9] Chalaire K. C. Kim T. K. Garcia-Rodriguez H. Mulenga A. (2011). *Amblyomma americanum* (L.) (*Acari*: *Ixodidae*) tick salivary gland serine protease inhibitor (serpin) 6 is secreted into tick saliva during tick feeding. *J. Exp. Biol.* 214 665–673. 10.1242/jeb.052076 21270316 PMC3027472

[B10] Cho K. Ishiwata T. Uchida E. Nakazawa N. Korc M. Naito Z. (2007). Enhanced expression of keratinocyte growth factor and its receptor correlates with venous invasion in pancreatic cancer. *Am. J. Pathol.* 170 1964–1974. 10.2353/ajpath.2007.060935 17525264 PMC1899460

[B11] Crispell G. Commins S. P. Archer-Hartman S. A. Choudhary S. Dharmarajan G. Azadi P. (2019). Discovery of alpha-gal-containing antigens in north american tick species believed to induce red meat allergy. *Front. Immunol.* 10:1056. 10.3389/fimmu.2019.01056 31156631 PMC6533943

[B12] de la Fuente J. (2018). Controlling ticks and tick-borne diseases.looking forward. *Ticks Tick Borne Dis.* 9 1354–1357. 10.1016/j.ttbdis.2018.04.001 29656834

[B13] Feng G. Hao D. Chai J. (2014). Processing of CXCL12 impedes the recruitment of endothelial progenitor cells in diabetic wound healing. *FEBS J.* 281 5054–5062. 10.1111/febs.13043 25211042

[B14] Fevrier B. Raposo G. (2004). Exosomes: endosomal-derived vesicles shipping extracellular messages. *Curr. Opin. Cell Biol.* 16 415–421. 10.1016/j.ceb.2004.06.003 15261674

[B15] Gillitzer R. (1996). [Role of chemokines in wound healing of human skin]. *Zentralbl. Chir.* 121(Suppl.) 29–30. 8967229

[B16] Gillitzer R. Toksoy A. Voss A. (2000). [Role of chemokines in human skin wound healing]. *Zentralbl. Chir.* 125(Suppl. 1) 56–59. 10929648

[B17] Glatz M. Means T. Haas J. Steere A. C. Mullegger R. R. (2017). Characterization of the early local immune response to Ixodes ricinus tick bites in human skin. *Exp. Dermatol.* 26 263–269. 10.1111/exd.13207 27623398 PMC5342933

[B18] Hajnicka V. Kocakova P. Slavikova M. Slovak M. Gasperik J. Fuchsberger N. (2001). Anti-interleukin-8 activity of tick salivary gland extracts. *Parasite Immunol.* 23 483–489. 10.1046/j.1365-3024.2001.00403.x 11589777

[B19] Hajnicka V. Vancova I. Kocakova P. Slovak M. Gasperik J. Slavikova M. (2005). Manipulation of host cytokine network by ticks: a potential gateway for pathogen transmission. *Parasitol* 130 333–342. 10.1017/s0031182004006535 15796016

[B20] Hajnicka V. Vancova-Stibraniova I. Slovak M. Kocakova P. Nuttall P. A. (2011). Ixodid tick salivary gland products target host wound healing growth factors. *Int. J. Parasitol.* 41 213–223. 10.1016/j.ijpara.2010.09.005 20934428

[B21] Han H. Roan F. Ziegler S. F. (2017). The atopic march: current insights into skin barrier dysfunction and epithelial cell-derived cytokines. *Immunol. Rev.* 278 116–130. 10.1111/imr.12546 28658558 PMC5492959

[B22] Hanel K. H. Cornelissen C. Luscher B. Baron J. M. (2013). Cytokines and the skin barrier. *Int. J. Mol. Sci.* 14 6720–6745. 10.3390/ijms14046720 23531535 PMC3645662

[B23] Hermance M. E. Thangamani S. (2015). Tick saliva enhances powassan virus transmission to the host, influencing its dissemination and the course of disease. *J. Virol.* 89 7852–7860. 10.1128/JVI.01056-15 25995246 PMC4505606

[B24] Ibelli A. M. Kim T. K. Hill C. C. Lewis L. A. Bakshi M. Miller S. (2014). A blood meal-induced *Ixodes scapularis* tick saliva serpin inhibits trypsin and thrombin, and interferes with platelet aggregation and blood clotting. *Int. J. Parasitol.* 44 369–379. 10.1016/j.ijpara.2014.01.010 24583183 PMC4089096

[B25] Imamura S. da Silva Vaz Junior I. Sugino M. Ohashi K. Onuma M. (2005). A serine protease inhibitor (serpin) from *Haemaphysalis longicornis* as an anti-tick vaccine. *Vaccine* 23 1301–1311. 10.1016/j.vaccine.2004.08.041 15652673

[B26] Jiang W. G. Sanders A. J. Ruge F. Harding K. G. (2012). Influence of interleukin-8 (IL-8) and IL-8 receptors on the migration of human keratinocytes, the role of PLC-gamma and potential clinical implications. *Exp. Ther. Med.* 3 231–236. 10.3892/etm.2011.402 22969874 PMC3438606

[B27] Kamata M. Tada Y. Uratsuji H. Kawashima T. Asano Y. Sugaya M. (2011). Semaphorin 7A on keratinocytes induces interleukin-8 production by monocytes. *J. Dermatol. Sci.* 62 176–182. 10.1016/j.jdermsci.2011.02.004 21524887

[B28] Kang J. Perry J. K. Pandey V. Fielder G. C. Mei B. Qian P. X. (2009). Artemin is oncogenic for human mammary carcinoma cells. *Oncogene* 28 2034–2045. 10.1038/onc.2009.66 19363524

[B29] Karim S. Essenberg R. C. Dillwith J. W. Tucker J. S. Bowman A. S. Sauer J. R. (2002). Identification of SNARE and cell trafficking regulatory proteins in the salivary glands of the lone star tick, *Amblyomma americanum* (L.). *Insect. Biochem. Mol. Biol.* 32 1711–1721. 12429123 10.1016/s0965-1748(02)00111-x

[B30] Karim S. Singh P. Ribeiro J. M. (2011). A deep insight into the sialotranscriptome of the gulf coast tick, *Amblyomma maculatum*. *PLoS One* 6:e28525. 10.1371/journal.pone.0028525 22216098 PMC3244413

[B31] Kawane K. Tanaka H. Kitahara Y. Shimaoka S. Nagata S. (2010). Cytokine-dependent but acquired immunity-independent arthritis caused by DNA escaped from degradation. *Proc. Natl. Acad. Sci. U.S.A.* 107 19432–19437. 10.1073/pnas.1010603107 20974942 PMC2984163

[B32] Kazimirova M. Thangamani S. Bartikova P. Hermance M. Holikova V. Stibraniova I. (2017). Tick-borne viruses and biological processes at the tick-host-virus interface. *Front. Cell. Infect. Microbiol.* 7:339. 10.3389/fcimb.2017.00339 28798904 PMC5526847

[B33] Kim B. Choi Y. E. Kim H. S. (2014). Eruca sativa and its flavonoid components, quercetin and isorhamnetin, improve skin barrier function by activation of peroxisome proliferator-activated receptor (PPAR)-alpha and suppression of inflammatory cytokines. *Phytother. Res.* 28 1359–1366. 10.1002/ptr.5138 24610745

[B34] Kim K. H. Chung W. S. Kim Y. Kim K. S. Lee I. S. Park J. Y. (2015). Transcriptomic analysis reveals wound healing of morus alba root extract by up-regulating keratin filament and CXCL12/CXCR4 signaling. *Phytother. Res.* 29 1251–1258. 10.1002/ptr.5375 26014513

[B35] Kotal J. Langhansova H. Lieskovska J. Andersen J. F. Francischetti I. M. Chavakis T. (2015). Modulation of host immunity by tick saliva. *J. Proteomics* 128 58–68. 10.1016/j.jprot.2015.07.005 26189360 PMC4619117

[B36] Labuda M. Austyn J. M. Zuffova E. Kozuch O. Fuchsberger N. Lysy J. (1996). Importance of localized skin infection in tick-borne encephalitis virus transmission. *Virology* 219 357–366. 10.1006/viro.1996.0261 8638401

[B37] Labuda M. Randolph S. E. (1999). Survival strategy of tick-borne encephalitis virus: cellular basis and environmental determinants. *Zentralbl. Bakteriol.* 289 513–524. 10652718 10.1016/s0934-8840(99)80005-x

[B38] Leitner W. W. Costero-Saint Denis A. Wali T. (2011). Immunological consequences of arthropod vector-derived salivary factors. *Eur. J. Immunol.* 41 3396–3400. 10.1002/eji.201190075 22125007

[B39] Lewis L. A. Radulovic Z. M. Kim T. K. Porter L. M. Mulenga A. (2015). Identification of 24h *Ixodes scapularis* immunogenic tick saliva proteins. *Ticks. Tick. Borne. Dis.* 6 424–434. 10.1016/j.ttbdis.2015.03.012 25825233 PMC4415496

[B40] Ludwig A. K. Giebel B. (2012). Exosomes: small vesicles participating in intercellular communication. *Int. J. Biochem. Cell. Biol.* 44 11–15. 10.1016/j.biocel.2011.10.005 22024155

[B41] Miyamoto Y. Suyama T. Yashita T. Akimaru H. Kurata H. (2007). Bone marrow subpopulations contain distinct types of endothelial progenitor cells and angiogenic cytokine-producing cells. *J. Mol. Cell. Cardiol.* 43 627–635. 10.1016/j.yjmcc.2007.08.001 17900610

[B42] Mulenga A. Khumthong R. Chalaire K. C. (2009). *Ixodes scapularis* tick serine proteinase inhibitor (serpin) gene family; annotation and transcriptional analysis. *BMC Genomics* 10:217. 10.1186/1471-2164-10-217 19435496 PMC2689274

[B43] Neelakanta G. Sultana H. (2015). Transmission-blocking vaccines: focus on anti-vector vaccines against tick-borne diseases. *Arch. Immunol. Ther. Exp.* 63 169–179. 10.1007/s00005-014-0324-8 25503555 PMC4429137

[B44] Neelakanta G. Sultana H. Fish D. Anderson J. F. Fikrig E. (2010). Anaplasma phagocytophilum induces *Ixodes scapularis* ticks to express an antifreeze glycoprotein gene that enhances their survival in the cold. *J. Clin. Invest.* 120 3179–3190. 10.1172/JCI42868 20739755 PMC2929727

[B45] Nuttall P. A. (2019). Wonders of tick saliva. *Ticks. Tick. Borne. Dis.* 10 470–481. 10.1016/j.ttbdis.2018.11.005 30459085

[B46] Nuttall P. A. Labuda M. (2003). Dynamics of infection in tick vectors and at the tick-host interface. *Adv. Virus. Res.* 60 233–272. 14689696 10.1016/s0065-3527(03)60007-2

[B47] Nuttall P. A. Labuda M. (2004). Tick-host interactions: saliva-activated transmission. *Parasitol* 129(Suppl.) S177–S189. 10.1017/s0031182004005633 15938511

[B48] Nuttall P. A. Paesen G. C. Lawrie C. H. Wang H. (2000). Vector-host interactions in disease transmission. *J. Mol. Microbiol. Biotechnol.* 2 381–386. 11075909

[B49] Ogawa Y. Miura Y. Harazono A. Kanai-Azuma M. Akimoto Y. Kawakami H. (2011). Proteomic analysis of two types of exosomes in human whole saliva. *Biol. Pharm. Bull.* 34 13–23. 21212511 10.1248/bpb.34.13

[B50] Patrick C. D. Hair J. A. (1975). Laboratory rearing procedures and equipment for multi-host ticks (*Acarina*: *Ixodidae*). *J. Med. Entomol.* 12 389–390. 10.1093/jmedent/12.3.389 1181449

[B51] Pongprayoon P. Niramitranon J. Kaewhom P. Kaewmongkol S. Suwan E. Stich R. W. (2019). Dynamic and structural insights into tick serpin from Ixodes ricinus. *J. Biomol. Struct. Dyn.* 38 2296–2303. 10.1080/07391102.2019.1630003 31215334

[B52] Prevot P. P. Adam B. Boudjeltia K. Z. Brossard M. Lins L. Cauchie P. (2006). Anti-hemostatic effects of a serpin from the saliva of the tick Ixodes ricinus. *J. Biol. Chem.* 281 26361–26369. 10.1074/jbc.M604197200 16672226

[B53] Prevot P. P. Beschin A. Lins L. Beaufays J. Grosjean A. Bruys L. (2009). Exosites mediate the anti-inflammatory effects of a multifunctional serpin from the saliva of the tick Ixodes ricinus. *FEBS J.* 276 3235–3246. 10.1111/j.1742-4658.2009.07038.x 19438720

[B54] Radulovic Z. M. Mulenga A. (2017). Heparan sulfate/heparin glycosaminoglycan binding alters inhibitory profile and enhances anticoagulant function of conserved *Amblyomma americanum* tick saliva serpin 19. *Insect. Biochem. Mol. Biol.* 80 1–10. 10.1016/j.ibmb.2016.11.002 27845251 PMC5214524

[B55] Regev-Rudzki N. Wilson D. W. Carvalho T. G. Sisquella X. Coleman B. M. Rug M. (2013). Cell-cell communication between malaria-infected red blood cells via exosome-like vesicles. *Cell* 153 1120–1133. 10.1016/j.cell.2013.04.029 23683579

[B56] Rennekampff H. O. Hansbrough J. F. Kiessig V. Dore C. Sticherling M. Schroder J. M. (2000). Bioactive interleukin-8 is expressed in wounds and enhances wound healing. *J. Surg. Res.* 93 41–54. 10.1006/jsre.2000.5892 10945942

[B57] Restivo T. E. Mace K. A. Harken A. H. Young D. M. (2010). Application of the chemokine CXCL12 expression plasmid restores wound healing to near normal in a diabetic mouse model. *J. Trauma.* 69 392–398. 10.1097/TA.0b013e3181e772b0 20699749

[B58] Ribeiro J. M. C. Zeidner N. Ledin K. Dolan M. C. Mather T. N. (2004). How much pilocarpine contaminates pilocarpine-induced tick saliva? *Med. Vet. Entomol.* 18 20–24. 15009442 10.1111/j.0269-283x.2003.0469.x

[B59] Roy A. Attarha S. Weishaupt H. Edqvist P. H. Swartling F. J. Bergqvist M. (2017). Serglycin as a potential biomarker for glioma: association of serglycin expression, extent of mast cell recruitment and glioblastoma progression. *Oncotarget* 8 24815–24827. 10.18632/oncotarget.15820 28445977 PMC5421891

[B60] Scholl D. C. Embers M. E. Caskey J. R. Kaushal D. Mather T. N. Buck W. R. (2016). Immunomodulatory effects of tick saliva on dermal cells exposed to *Borrelia burgdorferi*, the agent of Lyme disease. *Parasit Vectors.* 9:394. 10.1186/s13071-016-1638-7 27391120 PMC4938952

[B61] Schutyser E. Su Y. Yu Y. Gouwy M. Zaja-Milatovic S. Van Damme J. (2007). Hypoxia enhances CXCR4 expression in human microvascular endothelial cells and human melanoma cells. *Eur. Cytokine. Netw.* 18 59–70. 10.1684/ecn.2007.0087 17594938 PMC2665278

[B62] Sherman M. B. Freiberg A. N. Holbrook M. R. Watowich S. J. (2009). Single-particle cryo-electron microscopy of Rift Valley fever virus. *Virology* 387 11–15. 10.1016/j.virol.2009.02.038 19304307 PMC2673237

[B63] Sherman M. B. Guenther R. H. Tama F. Sit T. L. Brooks C. L. Mikhailov A. M. (2006). Removal of divalent cations induces structural transitions in red clover necrotic mosaic virus, revealing a potential mechanism for RNA release. *J. Virol.* 80 10395–10406. 10.1128/JVI.01137-06 16920821 PMC1641784

[B64] Strausberg R. L. Camargo A. A. Riggins G. J. Schaefer C. F. de Souza S. J. Grouse L. H. (2002). An international database and integrated analysis tools for the study of cancer gene expression. *Pharmacogenomics. J.* 2 156–164. 10.1038/sj.tpj.6500103 12082587

[B65] Sugino M. Imamura S. Mulenga A. Nakajima M. Tsuda A. Ohashi K. (2003). A serine proteinase inhibitor (serpin) from ixodid tick *Haemaphysalis longicornis*; cloning and preliminary assessment of its suitability as a candidate for a tick vaccine. *Vaccine* 21 2844–2851. 12798626 10.1016/s0264-410x(03)00167-1

[B66] Sultana H. Foellmer H. G. Neelakanta G. Oliphant T. Engle M. Ledizet M. (2009). Fusion loop peptide of the West nile virus envelope protein is essential for pathogenesis and is recognized by a therapeutic cross-reactive human monoclonal antibody. *J. Immunol.* 183 650–660. 10.4049/jimmunol.0900093 19535627 PMC3690769

[B67] Sultana H. Neelakanta G. Foellmer H. G. Montgomery R. R. Anderson J. F. Koski R. A. (2012). Semaphorin 7A contributes to West Nile virus pathogenesis through TGF-beta1/Smad6 signaling. *J. Immunol.* 189 3150–3158. 10.4049/jimmunol.1201140 22896629 PMC3496209

[B68] Sultana H. Neelakanta G. Kantor F. S. Malawista S. E. Fish D. Montgomery R. R. (2010). Anaplasma phagocytophilum induces actin phosphorylation to selectively regulate gene transcription in *Ixodes scapularis* ticks. *J. Exp. Med.* 207 1727–1743. 10.1084/jem.20100276 20660616 PMC2916137

[B69] Takada K. Komine-Aizawa S. Hirohata N. Trinh Q. D. Nishina A. Kimura H. (2017). Poly I:C induces collective migration of HaCaT keratinocytes via IL-8. *BMC Immunol.* 18:19. 10.1186/s12865-017-0202-3 28438134 PMC5404316

[B70] Thery C. Amigorena S. Raposo G. Clayton A. (2006). Isolation and characterization of exosomes from cell culture supernatants and biological fluids. *Curr. Protoc. Cell Biol. Chapter* 3:22. 10.1002/0471143030.cb0322s30 18228490

[B71] Vagesjo E. Ohnstedt E. Mortier A. Lofton H. Huss F. Proost P. (2018). Accelerated wound healing in mice by on-site production and delivery of CXCL12 by transformed lactic acid bacteria. *Proc. Natl. Acad. Sci. U.S.A.* 115 1895–1900. 10.1073/pnas.1716580115 29432190 PMC5828606

[B72] Valenzuela J. G. (2004). Exploring tick saliva: from biochemistry to ‘sialomes’ and functional genomics. *Parasitol* 129(Suppl.) S83–S94. 10.1017/s0031182004005189 15938506

[B73] Vella L. J. Scicluna B. J. Cheng L. Bawden E. G. Masters C. L. Ang C. S. (2017). A rigorous method to enrich for exosomes from brain tissue. *J. Extracell. Vesicles.* 6:1348885. 10.1080/20013078.2017.1348885 28804598 PMC5533148

[B74] Vora A. Taank V. Dutta S. M. Anderson J. F. Fish D. Sonenshine D. E. (2017). Ticks elicit variable fibrinogenolytic activities upon feeding on hosts with different immune backgrounds. *Sci. Rep.* 7:44593. 10.1038/srep44593 28300174 PMC5353578

[B75] Vora A. Zhou W. Londono-Renteria B. Woodson M. Sherman M. B. Colpitts T. M. (2018). Arthropod EVs mediate dengue virus transmission through interaction with a tetraspanin domain containing glycoprotein Tsp29Fb. *Proc. Natl. Acad. Sci. U.S.A.* 115 E6604–E6613. 10.1073/pnas.1720125115 29946031 PMC6048473

[B76] Wikel S. K. (1996a). Host immunity to ticks. *Annu. Rev. Entomol.* 41 1–22. 10.1146/annurev.en.41.010196.000245 8546443

[B77] Wikel S. K. (1996b). Tick modulation of host cytokines. *Exp. Parasitol.* 84 304–309. 10.1006/expr.1996.0118 8932782

[B78] Wikel S. K. (2018). Tick-host-pathogen systems immunobiology: an interactive trio. *Front. Biosci.* 23:265–283. 10.2741/4590 28930546

[B79] Wikel S. K. Whelen A. C. (1986). Ixodid-host immune interaction. Identification and characterization of relevant antigens and tick-induced host immunosuppression. *Vet. Parasitol.* 20 149–174. 2422805 10.1016/0304-4017(86)90098-1

[B80] Willis C. M. Nicaise A. M. Menoret A. Ryu J. K. Mendiola A. S. Jellison E. R. (2019). Extracellular vesicle fibrinogen induces encephalitogenic CD8+ T cells in a mouse model of multiple sclerosis. *Proc. Natl. Acad. Sci. U.S.A.* 116 10488–10493. 10.1073/pnas.1816911116 31068461 PMC6535008

[B81] Xie S. Macedo P. Hew M. Nassenstein C. Lee K. Y. Chung K. F. (2009). Expression of transforming growth factor-beta (TGF-beta) in chronic idiopathic cough. *Respir. Res.* 10:40. 10.1186/1465-9921-10-40 19463161 PMC2688489

[B82] Zhou W. Woodson M. Neupane B. Bai F. Sherman M. B. Choi K. H. (2018). Exosomes serve as novel modes of tick-borne flavivirus transmission from arthropod to human cells and facilitates dissemination of viral RNA and proteins to the vertebrate neuronal cells. *PLoS Pathog.* 14:e1006764. 10.1371/journal.ppat.1006764 29300779 PMC5754134

[B83] Zhou W. Woodson M. Sherman M. B. Neelakanta G. Sultana H. (2019). Exosomes mediate Zika virus transmission through SMPD3 neutral Sphingomyelinase in cortical neurons. *Emerg. Microbes. Infect.* 8 307–326. 10.1080/22221751.2019.1578188 30866785 PMC6455149

