# Supplementary figures and images for: Discovery of Exosomes From Tick Saliva and Salivary Glands Reveals Therapeutic Roles for CXCL12 and IL-8 in Wound Healing at the Tick–Human Skin Interface
Source: Front Cell Dev Biol. 2020 Jul 16;8:554. doi: 10.3389/fcell.2020.00554 (PMC7378379; doi:10.3389/fcell.2020.00554)

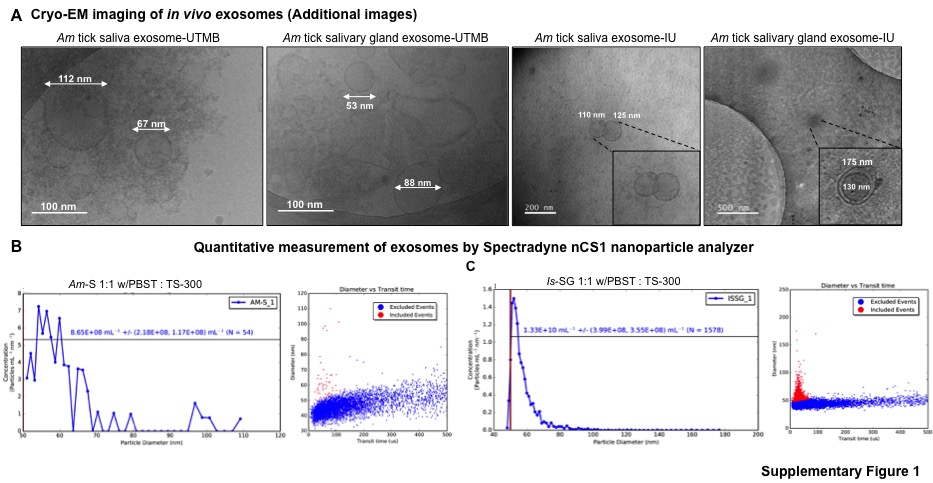

Supplement: FIGURE S1 — Cryo-EM imaging and measurement of in vivo exosomes derived from Am saliva and salivary glands. (A) Additional cryo-EM (electron microscopy) images collected from two independent institutions (University of Texas Medical Branch; UTMB and Indiana University; IU) showing exosomes isolated from Am saliva or salivary gland tissues. Scale bar indicates 100 nm (UTMB), or 200 or 500 nm (IU), respectively. For better comparison, scales have been provided close to individual exosomes. For images from IU on right side, insets show detailed structure of exosomes from the field. Measurement of diameters shows heterogeneous population of in vivo exosomes from saliva or salivary glands. Particle diameter measurement (in nm) analysis against the concentration of particles to determine the exosome sizes from Am saliva (indicated as Am-S) (B) or Is salivary gland-derived exosomes (represented as Is-SG) (C) is shown. N represents number of particles determined or counted using the TS-300 filter that measured 50–300 nm particle sizes. Scatter plot graphs on right in both (B) and (C) shows number of particles counted during the transit time (indicated as N) in Spectradyne nCS1 nanoparticle analyzer. Am represents (Amblyomma maculatum) and Is indicates (Ixodes scapularis) ticks. [file Image_1.JPEG]

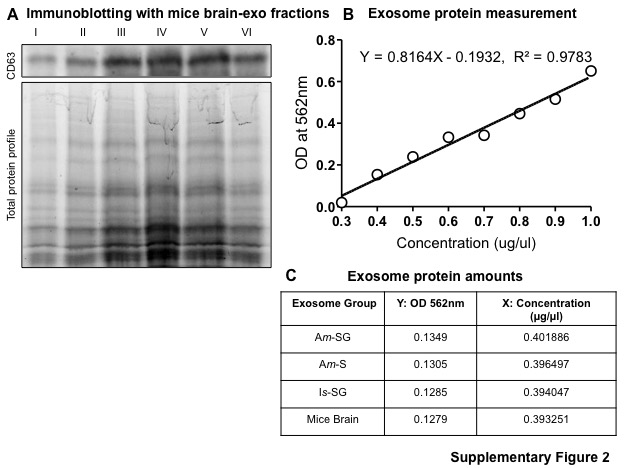

Supplement: FIGURE S2 — Detection of CD63 from brain tissue-derived exosomal fractions and estimation of proteins from tick exosomes. (A) Immunoblotting analysis showing detection of exosomal enriched marker CD63 in six different exosomal fractions prepared from C57/BL6 wild type mice brain tissue. Total protein profile is shown from the stain free gel image that serves as loading control. (B,C) BCA measurement assay showing the estimated protein amounts at an optical density of 562 nm (shown in Y-axis) and concentration of tick exosomal proteins (in μg/μl). The R2 value is shown for the standards. [file Image_2.JPEG]

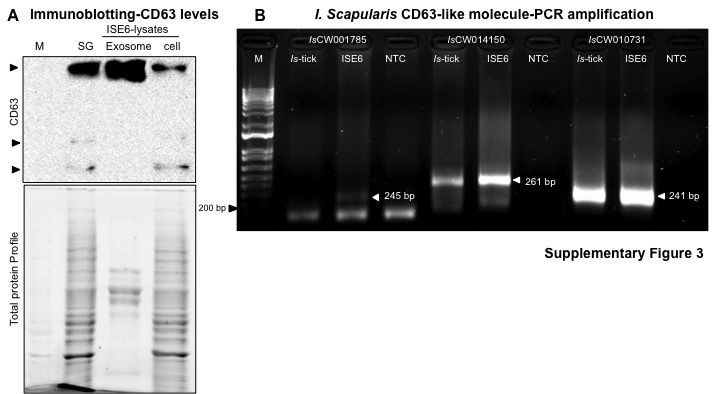

Supplement: FIGURE S3 — Enhanced CD63 levels in tick cell-derived exosomes and amplification of Is CD63-like molecules. (A) Immunoblotting analysis showing detection of exosomal enriched marker CD63 in Am whole salivary gland lysates, tick cell-derived exosomes, and whole tick cell lysates. Arrowheads indicate the endogenous (lower) and glycosylated (middle and upper) protein in salivary gland and ISE6 cell and exosomal lysates. Total protein profile is shown from the stain free gel image that serves as loading control. M indicate protein ladder and SG denotes salivary glands. (B) PCR amplification of CD63-like genes from Is unfed female ticks or ISE6 cells cDNA is shown. Three different fragments were amplified, and bands of approximately 245, 261 and 241 bp (denoted by arrowheads) were detected on 1% agarose gel for ISE6 cells. Is unfed female ticks showed amplified product for two CD63-like molecules (IsCW014150, and IsCW010731). NTC denotes no template control, and NTC is shown for respective primer pairs. M represents DNA ladder and Is shows Ixodes scapularis. Arrowhead indicates the 200 bp reference bands in DNA ladder. [file Image_3.JPEG]

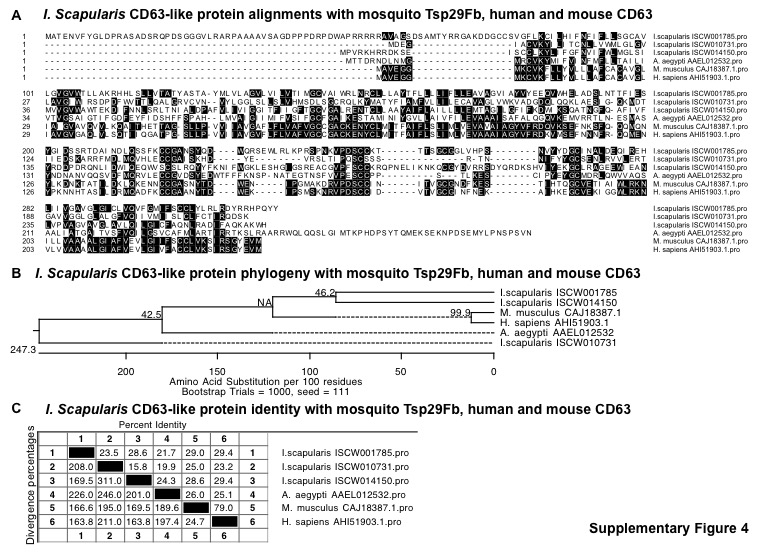

Supplement: FIGURE S4 — Alignment, phylogenetic analysis and identity of Is CD63-like proteins. (A) Deduced Is CD63-like amino acid sequence alignments (with other orthologs) using ClustalW program in DNASTAR Lasergene is shown. Residues that match are shaded in black color. GenBank accession numbers for M. musculus and H. sapiens CD63 sequences are shown. VectorBase accession numbers for three of the Is CD63-like proteins, and A. aegypti Tsp29Fb are provided. Total length of the amino acid sequence is provided at left end of each sequence. (B) Phylogenetic analysis was performed in DNASTAR by ClustalW slow/accurate alignment method using Gonnet as default value for protein weight matrix. Scale at the bottom denotes amino acid substitutions per 100 amino acid residues. Bootstrap and seed numbers are provided. (C) Percent identity (horizontally above black boxed diagonal) and divergence (vertically below black boxed diagonal) of Is CD63-like nucleotide sequence in comparison to A. aegypti Tsp29Fb, M. musculus and H. sapiens CD63 sequences is shown. [file Image_4.JPEG]

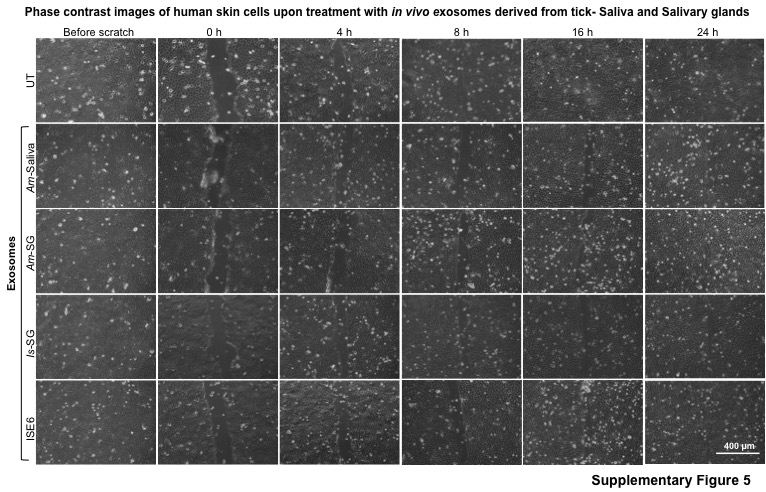

Supplement: FIGURE S5 — Exosomes derived from tick saliva, salivary glands, or ISE6 cells delay wound closure and repair in skin keratinocytes. Phase contrast images of HaCaT cells monolayers treated with 20 μl of exosomal-pooled fractions (1–6) from either Am saliva or salivary glands or Is salivary glands or ISE6 cells for 24 h is shown. Images were obtained before any treatments and shown as before scratch. Scratch generated cell images before treatment with tick exosomes are shown as 0 h. Representative images are shown for each time points (of 0, 4, 8, 16, and 24 h) post tick exosome-treatments. Images from time points of 0, 8, 16, and 24 h are previously shown in Figure 2 and are repeated in this figure for better comparison with inclusion of before scratch and 4 h time point group. HaCaT cell monolayers maintained as untreated (UT) group serve as control. Images were obtained using EVOS FL system and 10X magnification. Scale bar indicates 400 μm for each image per group/time point. Am represents (Amblyomma maculatum), Is indicates (Ixodes scapularis) and ISE6 denotes Is-derived cell line. Two-way ANOVA analysis is shown in Supplementary Table S1A. [file Image_5.JPEG]

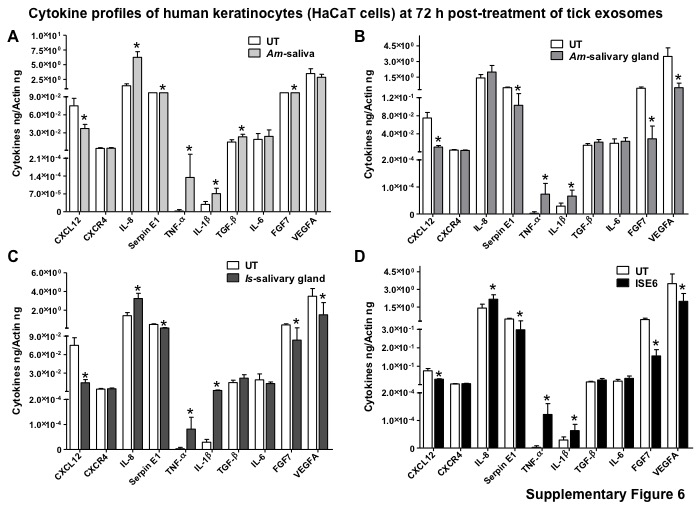

Supplement: FIGURE S6 — Human skin cells cytokine analysis revealed differential regulation by tick saliva, salivary gland, or ISE6 cell-derived exosomes. QRT-PCR analysis showing levels of different cytokines/chemokines (CXCL12, CXCR4, IL-8, Serpin E1, TNF-α, IL-1β, TGF-β, IL-6, FGF-7 and VEGFA) from HaCaT cells (A–D) treated (for 72 h) with exosomes derived from Am saliva or salivary glands, Is salivary glands or ISE6 cells. Transcript levels were compared to the levels of the cytokine or chemokine levels in untreated (UT) HaCaT cells. Cytokine levels were normalized to human beta-actin, respectively. Asterisk indicates significance (P < 0.05) in comparison to respective untreated controls. P-value determined by Student’s two-tailed t-test is shown. [file Image_6.JPEG]

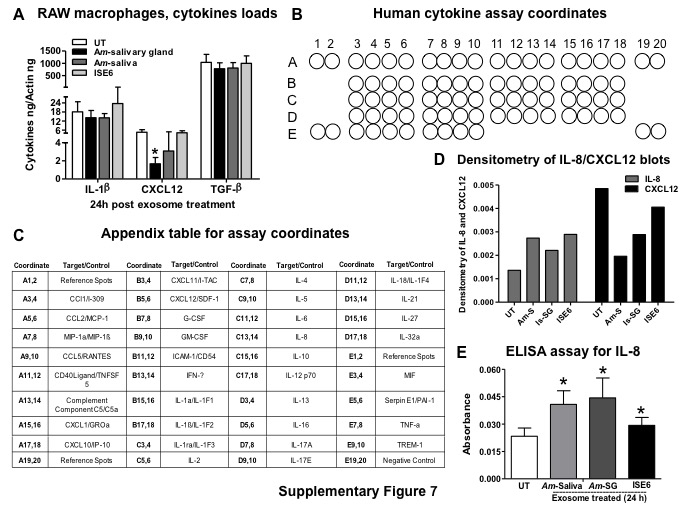

Supplement: FIGURE S7 — Cytokine expression in mouse macrophages, protein array profile layouts and detection of IL-8 upregulation by ELISA. (A) QRT-PCR analysis showing expression of IL-1β, TGF-β and CXCL12 from mouse macrophages (RAW264.7 cell line) treated (for 24 h) with exosomes from Am saliva or salivary glands, or ISE6 cells. Each cytokine load is compared to its respective untreated control group. UT indicates untreated. Transcript levels in RAW 264.7 cells were normalized to mouse beta-actin, respectively. (B) Layout of human cytokine assay coordinates spotted on four of the nitrocellulose membranes purchased from R&D systems is shown. (C) Appendix table for assay coordinates showing the details of cytokines/chemokines spotted in duplicate on the nitrocellulose membrane is provided from the vendor’s website. The Reference proteins as positive control are spotted on membranes at A1, 2; E1, 2 and A19, 20 whereas E19, 20 were negative controls for the assay. (D) Densitometry analysis showing differences in secreted protein levels of IL-8 or CXCL12 in comparison to the untreated (UT) control. Am represents (Amblyomma maculatum), Is indicates (Ixodes scapularis) and ISE6 denotes Is-derived cell line. (E) ELISA assay showing IL-8 expression in cell culture supernatants collected from HaCaT cells treated (24 h) with exosomes from Am saliva or salivary glands, or ISE6 cells. SG indicates salivary glands. Asterisk indicates significance (P < 0.05) in comparison to respective untreated controls. P-value determined by Student’s two-tailed t-test is shown. [file Image_7.JPEG]

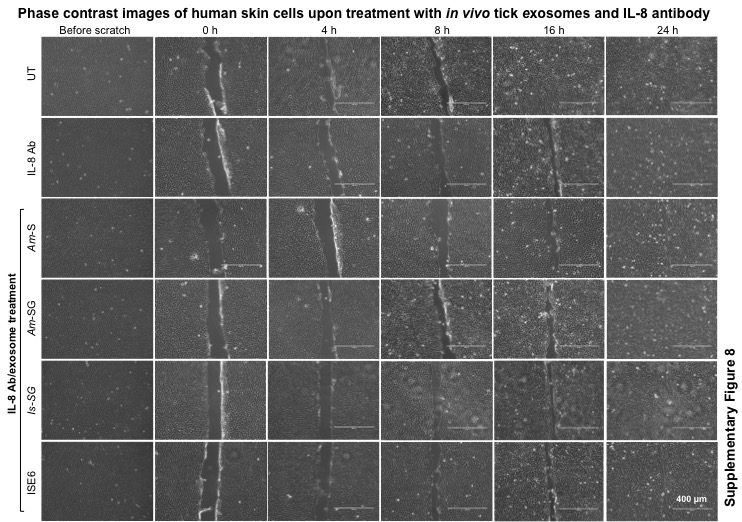

Supplement: FIGURE S8 — Blocking of IL-8 via antibody followed by treatment with exosomes derived from tick saliva, salivary glands, or ISE6 cells further delays wound closure and repair. Phase contrast images of HaCaT cell monolayers treated with IL-8 antibody (2 μg) for 12 h, followed by treatments with 20 μl of exosomal-pooled fractions (1-6) from either Am saliva, or salivary glands or Is salivary glands or ISE6 cells for 24 h is shown. Images of HaCaT cell monolayers collected before any treatments served as before scratch internal control. Scratches were generated and images collected immediately after scratches as 0 h, followed by treatment (for 24 h) of HaCaT cells with tick exosomes is shown. Representative images are shown for each time points (of 0, 4, 8, 16, and 24 h) post tick exosome-treatments. Images from time points of 0, 8, 16, and 24 h are previously shown in Figure 5D, and repeated in this figure for comparison with before scratch and 4 h group. HaCaT cell monolayers that were maintained as untreated (UT) served as control. Images were obtained using EVOS FL system and 10X magnification. Scale bar indicates 400 μm for each image/group/time point. Am represents (Amblyomma maculatum), Is indicates (Ixodes scapularis) and ISE6 denotes Is-derived cell line. Two-way ANOVA analysis is shown in Supplementary Table S1B. [file Image_8.JPEG]

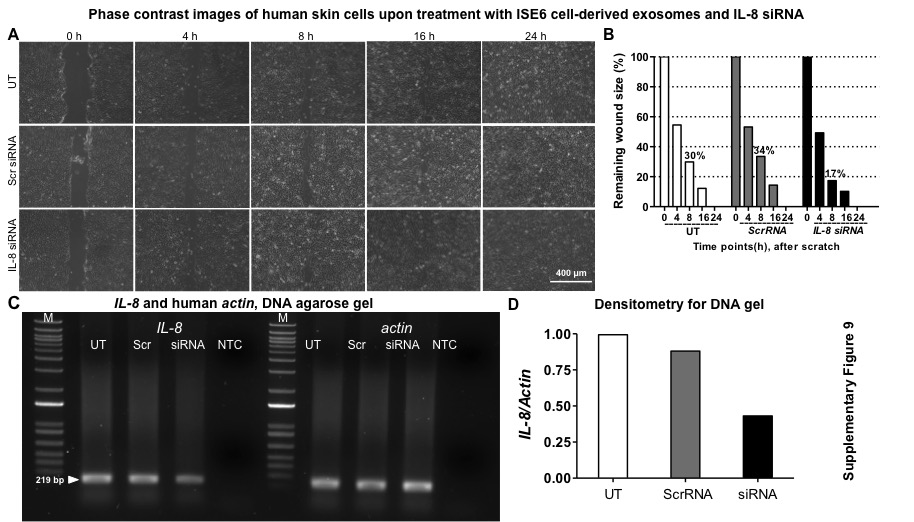

Supplement: FIGURE S9 — Silencing of IL-8 via siRNA followed by treatment with exosomes derived from tick cells. (A) Phase contrast images of HaCaT cell monolayers treated with IL-8 siRNA (∼1 μg) for 24 h, followed by scratches and treatments with 20 μl of exosomal-pooled fractions (1–6) from ISE6 cells for 24 h is shown. Images were collected immediately after scratches as 0 h. Representative images are shown for each time points (of 0, 4, 8, 16, and 24 h) post tick exosome-treatments. Scrambled (Scr) siRNA group serve as control. HaCaT cell monolayers that were maintained as untreated (UT) served as internal control. Images were obtained using EVOS FL system and 10X magnification. Scale bar indicates 400 μm for each image/group/time point. (B) Quantitative measurement of percentages of remaining wound sizes at different time points of 0, 4, 8, 16, and 24 h are shown for IL-8 siRNA, or scrambled siRNA in combination with ISE6 cell-derived exosomes. Wounds at 0 h were considered as 100% for all groups, including IL-8 siRNA, or scrambled siRNA or untreated (UT) controls. Percentages for remaining wound sizes at 8 or 24 h posttreatment of ISE6 cell-derived exosomes are shown on bar graphs for comparison. Two-way ANOVA analysis is shown in Supplementary Table S2A. (C) PCR amplification and DNA agarose gel electrophoresis (1.2% gel) of HaCaT cells collected from wound healing assay showing transcripts of IL-8 in siRNA treated, scrambled, or untreated groups. The actin transcripts are shown as internal control. M indicate DNA ladder and arrowhead denotes an amplified product of 219 bp. (D) Densitometry analysis showing the reduction in IL-8 amplified product in siRNA treated group in comparison to the scrambled or untreated controls. [file Image_9.JPEG]

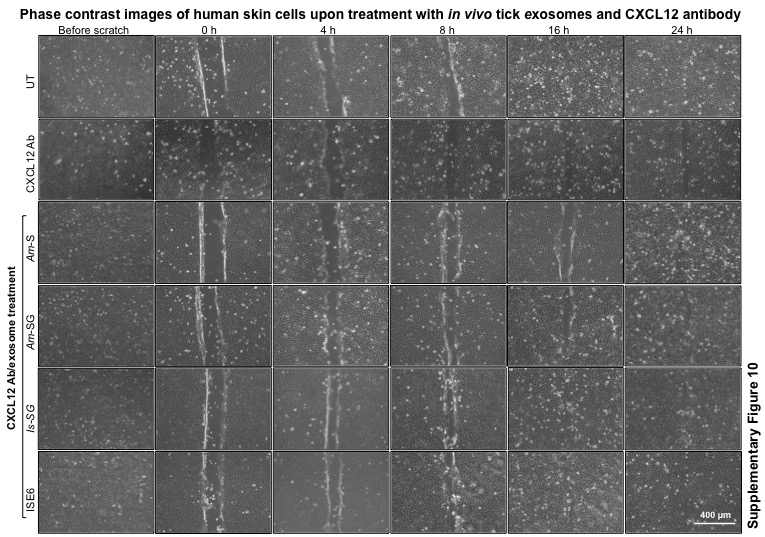

Supplement: FIGURE S10 — Blocking of CXCL12 followed by treatment with exosomes derived from tick saliva, salivary glands, or ISE6 cells further delays wound closure and repair. Phase contrast images of HaCaT cell monolayers treated with CXCL12 antibody (2 μg) for 12 h, followed by treatment with 20 μl of exosomal-pooled fractions (1–6) from either Am saliva, or salivary glands or Is salivary glands or ISE6 cells for 24 h is shown. Images of HaCaT cells before scratch or any treatments served as internal control group. Images collected immediately after scratches are considered as 0 h and shown for comparison. Scratches were generated and tick exosomes were treated for 24 h and representative images are shown for each time points (of 0, 4, 8, 16, and 24 h). Images from time points of 0, 8, 16, and 24 h are previously shown in Figure 6A, and repeated here for comparison with before scratch and 4 h time point groups. HaCaT cell monolayers that were maintained as untreated (UT) served as control group. Images were obtained using EVOS FL system and 10X magnification. Scale bar indicates 400 μm for each image per group/time point. Am represents (Amblyomma maculatum), Is indicates (Ixodes scapularis) and ISE6 denotes Is-derived cell line. Two-way ANOVA analysis is shown in Supplementary Table S1B. [file Image_10.JPEG]

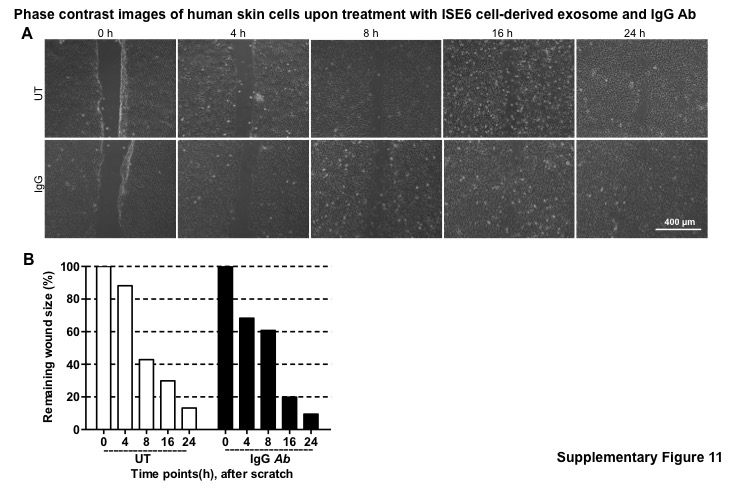

Supplement: FIGURE S11 — Treatment of isotype control IgG antibody did not interfere with wound healing process. (A) Phase contrast images of HaCaT cell monolayers treated with isotype control IgG antibody (2 μg) for 24 h, followed by scratches and treatments with 20 μl of exosomal-pooled fractions (1–6) from ISE6 cells for 24 h is shown. Images were collected immediately after scratches as 0 h. Representative images are shown for each time points (of 0, 4, 8, 16, and 24 h) post tick exosome treatments. Untreated (UT) group served as internal control. Images were obtained using EVOS FL system and 10X magnification. Scale bar indicates 400 μm for each image/group/time point. (B) Quantitative measurement of percentages of remaining wound sizes at different time points of 0, 4, 8, 16, and 24 h are shown for IgG isotype antibody group treated with ISE6 cell-derived exosomes or untreated control. Wounds at 0 h were considered as 100% for all groups, including IgG isotype antibody group or untreated (UT) control. Percentages for remaining wound sizes at 16 or 24 h posttreatment of ISE6 cell-derived exosomes are shown on bar graphs for comparison. Two-way ANOVA analysis is shown in Supplementary Table S2B. [file Image_11.JPEG]

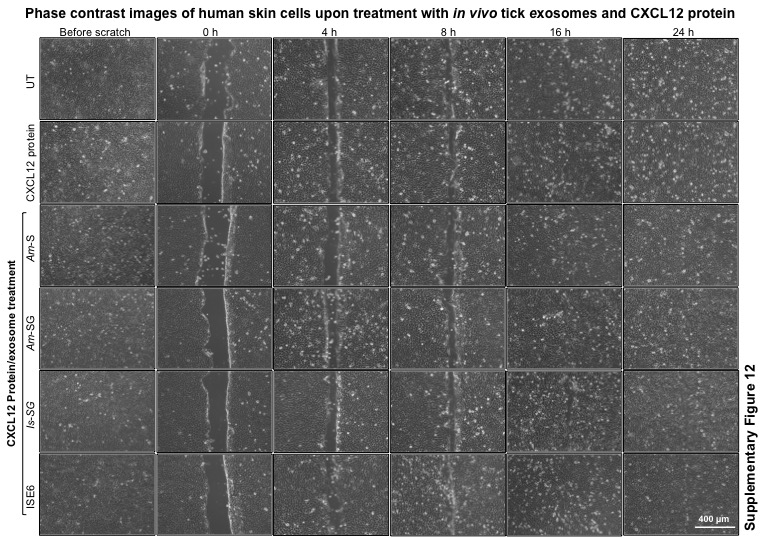

Supplement: FIGURE S12 — CXCL12 exogenous protein treatment restored delayed wound closure and repair. Images from purified GST-tagged CXCL12 protein (2 μg) treated on HaCaT cell monolayers for 4 h, then followed by treatments with 20 μl of exosomal-pooled fractions (1–6) from either Am saliva or salivary glands or Is salivary glands or ISE6 cells for 24 h are shown. Images of HaCaT cell monolayers before scratch served as internal control group. Images collected immediately after scratches and before treatment with tick exosomes are shown as 0 h. Representative phase contrast images are shown for each time points (of 0, 4, 8, 16, and 24 h) post tick exosome treatments. Images from time points of 0, 8, 16, and 24 h are previously shown in Figure 6C and repeated in this figure for comparison with before scratch and 4 h time point groups. HaCaT cell monolayers, that were maintained as untreated (UT) serve as control. Images were obtained using EVOS FL system and 10X magnification. Scale bar indicates 400 μm for each image per group/time point. Am represents (Amblyomma maculatum), Is indicates (Ixodes scapularis) and ISE6 denotes Is-derived cells. Two-way ANOVA analysis is shown in Supplementary Table S1B. [file Image_12.JPEG]

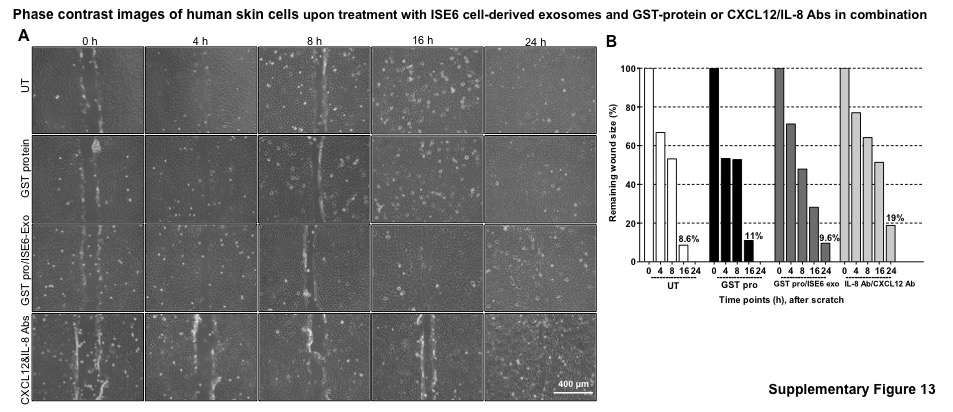

Supplement: FIGURE S13 — Treatment of GST purified protein does not influence tick exosome-mediated delay in wound closure and repair. (A) Purified GST protein alone (2 μg) treated on HaCaT cell monolayers for 4 h, followed by treatments with 20 μl of exosomal-pooled fractions (1–6) from ISE6 cells for 24 h is shown. Images from IL-8 and CXCL12 combination antibodies (2 μg, each) treatment is also shown as control. Images of HaCaT cell monolayers taken before scratch serve as control. Scratches were generated and images were collected immediately after scratches as 0 h. Representative phase contrast images are shown for each time points (of 0, 4, 8, 16, and 24 h) post tick exosome treatments. HaCaT cell monolayers, that were maintained as untreated (UT) serve as control. Images were obtained using EVOS FL system and 10X magnification. Scale bar indicates 400 μm for each image per group/time point. (B) Quantitative measurement of percentages of remaining wound sizes at different time points of 0, 4, 8, 16, and 24 h are shown for GST protein alone, or GST protein in combination with ISE6 cell-derived exosome treatment or treatments with combination of IL-8 and CXCL12 antibodies is shown. Wounds at 0 h were considered as 100% for all groups, including GST protein alone or untreated (UT) controls. Percentages for remaining wound sizes at 16 or 24 h posttreatment of ISE6 cell-derived exosomes are shown on bar graphs for comparison. Two-way ANOVA analysis is shown in Supplementary Table S2C. [file Image_13.JPEG]
