# Supplementary material for: Discovery of Exosomes From Tick Saliva and Salivary Glands Reveals Therapeutic Roles for CXCL12 and IL-8 in Wound Healing at the Tick–Human Skin Interface
Source: Front Cell Dev Biol. 2020 Jul 16;8:554. doi: 10.3389/fcell.2020.00554 (PMC7378379; doi:10.3389/fcell.2020.00554)
Supplement: TABLE S1 — Two-way ANOVA analysis for HaCaT cells incubated with ISE6 cell-derived exosome treatments and treatments with IL-8/CXCL12 Antibodies or CXCL12 protein. (A) Statistical significance is indicated as Yes or No below black-boxed diagonal and P-value for each comparison is indicated above black boxed diagonal. All significant and marginal significant values are in bold text. (B) Statistical significance was compared to mock-treated group. Time point posttreatment is shown. All significant and marginal significant values are in bold text. [file Table_1.pdf]

Table 1: Two-way ANOVA of Wound healing assay

A Two-way ANOVA significance (exosome treatment)

P value / significant	Un-treated	*Am*-Saliva exo	*Am*-SG exo	*Is*-SG exo	ISE6 exo
Un-treated		**0.038**	**0.041**	**0.027**	**0.083**
Am-Saliva exo	**Yes**		0.115	0.115	**0.041**
Am-SG exo	**Yes**	No		0.55	0.99
Is-SG exo	**Yes**	No	No		0.6
ISE6 exo	**Marginal**	**Yes**	No	No	

B Two-way ANOVA significance (Ab and Protein treatment)

Time point	4 H	8 H	16 H	24 H
P value / Group / Group	MOCK	MOCK	MOCK	MOCK
IL-8 Ab	**0.029**	0.976	0.678	0.328
CXCL12 Ab	0.636	0.497	0.821	0.638
CXCL12 Pro	**0.004**	**0.019**	**0.046**	**0.048**

* Note: Compared among exosome treated or un-treated groups (*Am*-Saliva, *Am*-SG, *Is*-SG and ISE6)

Table 2: Wound healing assay, ANOVA

A Two-way ANOVA for *IL-8* siRNA silencing Exp

Source of Variation	SS	df	MS	F	P-value	F crit
**Columns**	66.58231	2	33.29115	2.655388	**0.130481**	4.45897

* Note: Compared among un-treated, *IL-8* siRNA treated and *Scramble RNA* treated

B Two-way ANOVA for UT and *IgG* Ab treated

Source of Variation	SS	df	MS	F	P-value	F crit
**Rows**	24.52353	1	24.52353	0.250357	**0.6431**	7.708647

* Note: Compared between un-treated and *IgG* Ab treated

C Two-way ANOVA for UT and GST protein treated

Source of Variation	SS	df	MS	F	P-value	F crit
**Rows**	13.01246	1	13.01246	0.649662	**0.46542**	7.708647

* Note: Compared between un-treated and GST protein treated

Table 3. Oligonucleotides used in this study

Forward Primer (5'-3')	Reverse Primer (5'-3')	Human Gene
TCTGCAGCTCTGTGTGAAGGTGC	AACCCTCTGCACCCAGTTTTCCT	*IL-8*
CCGAGGCAGTCAGATCATCTT	AGCTGCCCCTCAGCTTGA	*TNF-alpha*
CACAAATCAGACGGCAGCACT	CATCGGGCGTGGTGAACTC	*Serpin E1*
CACTTCAGATAACTACACCG	ATCCAGACGCCAACATAGAC	*CXCR4*
CCCAGCATCTGCAAAGCTC	GTCAATGTACAGCTGCCGCA	*TGF-beta*
AGACAGCCACTCACCTCTTCAG	TTCTGCCAGTGCCTCTTTGCTG	*IL-6*
CTCAACACTCCAAACTGTGCCC	CTCCAGGTACTCCTGAATCCAC	*CXCL12*
TTGTGGCAATCAAAGGGGTG	CCTCCGTTGTGTGTCCATTTAGC	*FGF7*
GAGGAGGGCAGAATCATCACGAA	TGGTGAGGTTTGATCCGCATAA	*VEGFA*
AGCCTCGCCTTTGCCGA	CTGGTGCCTGGGGCG	*Actin*
**Forward Primer (5'-3')**	**Reverse Primer (5'-3')**	**Mouse Gene**
TTGGGCCTCAAAGGAAAGAA	TGGGTATTGCTTGGGATCCA	*IL-1 beta*
GACCGCAACAACGCCATCTA	GGCGTATCAGTGGGGGTCAG	*TGF-beta*
CCCTAGGCACCAGGGTGTGA	GGGGTGTTGAAGGTCTCAAACA	*Actin*
**Forward Primer (5'-3')**	**Reverse Primer (5'-3')**	**Tick Gene**
GCATCCTCCACATCTTCAACTTCA	CAGGAGCAGGAGGAAGGTGT	ISCW001785-RA
GCTCTGGGAAGAGTGTGCGT	CCTCCTGGCTTTTGAGTCCTCT	ISCW010731-RA
GGCAGGCATTTTGGGATTCA	GCAGCACGAGAAAGGGACA	ISCW014150-RA
